# Ascorbic acid metabolism and functions: A comparison of plants and mammals

**DOI:** 10.1016/j.freeradbiomed.2018.03.033

**Published:** 2018-07

**Authors:** Nicholas Smirnoff

**Affiliations:** Biosciences, College of Life and Environmental Sciences, University of Exeter, Geoffrey Pope Building, Stocker Road, Exeter EX4 4QD, UK

**Keywords:** 2-ODD, 2-oxolutarate-dependent dioxygenase, APX, ascorbate peroxidase, AO, ascorbate oxidase, DHA, dehydroascorbate (bicyclic), DHAR, dehydroascorbate reductase, GLUT, DHA transporter, GSH, glutathione, GSSG, glutathione disulfide, MDHA, monodehydroascorbate, MDHAR, monodehydroascorbate reductase, SOD, superoxide dismutase, SVCT, sodium-dependent ascorbate transporter, VDE, violaxanthin de-epoxidase, Ascorbic acid, Vitamin C, Hydrogen peroxide, Ascorbate peroxidase, Ascorbate oxidase, Gulonolactone oxidase, Galactonolactone dehydrogenase, Dehydroascorbate, Monodehydroascorbate, Dioxygenase, Iron reduction, Epigenetics, *vtc* mutants

## Abstract

Ascorbic acid is synthesised by eukaryotes, the known exceptions being primates and some other animal groups which have lost functional gulonolactone oxidase. Prokaryotes do not synthesise ascorbate and do not need an ascorbate supply, so the functions that are essential for mammals and plants are not required or are substituted by other compounds. The ability of ascorbate to donate electrons enables it to act as a free radical scavenger and to reduce higher oxidation states of iron to Fe^2+^. These reactions are the basis of its biological activity along with the relative stability of the resulting resonance stabilised monodehydroascorbate radical. The importance of these properties is emphasised by the evolution of at least three biosynthetic pathways and production of an ascorbate analogue, erythroascorbate, by fungi. The iron reducing activity of ascorbate maintains the reactive centre Fe^2+^ of 2-oxoglutarate-dependent dioxygenases (2-ODDs) thus preventing inactivation. These enzymes have diverse functions and, recently, the possibility that ascorbate status in mammals could influence 2-ODDs involved in histone and DNA demethylation thereby influencing stem cell differentiation and cancer has been uncovered. Ascorbate is involved in iron uptake and transport in plants and animals. While the above biochemical functions are shared between mammals and plants, ascorbate peroxidase (APX) is an enzyme family limited to plants and photosynthetic protists. It provides these organisms with increased capacity to remove H_2_O_2_ produced by photosynthetic electron transport and photorespiration. The Fe reducing activity of ascorbate enables hydroxyl radical production (pro-oxidant effect) and the reactivity of dehydroascorbate (DHA) and reaction of its degradation products with proteins (dehydroascorbylation and glycation) is potentially damaging. Ascorbate status influences gene expression in plants and mammals but at present there is little evidence that it acts as a specific signalling molecule. It most likely acts indirectly by influencing the redox state of thiols and 2-ODD activity. However, the possibility that dehydroascorbylation is a regulatory post-translational protein modification could be explored.

## Highlights

• The function and metabolism of ascorbate is compared between plants and mammals.
• Ascorbate readily reduces radicals and Fe/Cu.
• Ascorbate is synthesised by plants, protists and animals *via* different pathways.
• Fungi synthesise the analogue D-erythroascorbate.
• Plants and photosynthetic protists use ascorbate peroxidase to remove H_2_O_2_.

## Graphical abstract

**Figure:**
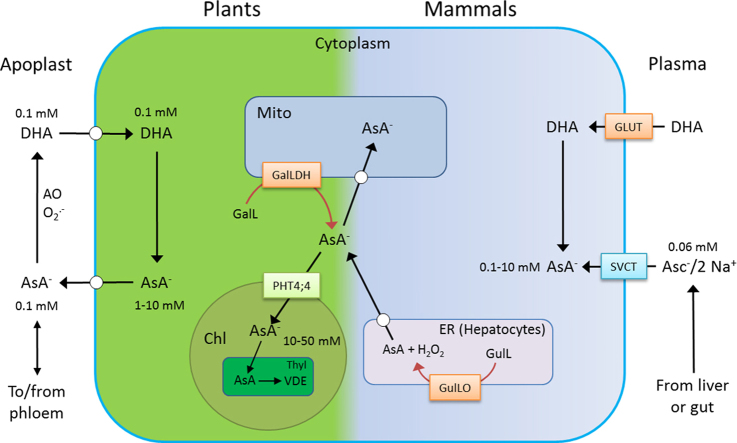

## 1 Introduction

Ascorbic acid (ascorbate, vitamin C) is a simultaneously well-known and surprisingly poorly-understood compound. It is synthesised only by eukaryotes and, to date, chemical analyses have not provided strong evidence for its synthesis by prokaryotes. A number of animal groups, including primates, lack ascorbate biosynthesis capacity due to loss of function mutations in the biosynthetic enzyme L-gulono-1,4-lactone oxidase [1] but according to genome sequence analysis biosynthesis capacity may be lacking in other protist groups [2]. There has been much speculation about the reasons for loss of ascorbate biosynthesis capacity but no easily testable hypotheses have emerged. It is essential for both plants and mammals but not for prokaryotes. This difference must reflect divergence between the radical removal and redox systems or the enzyme cofactor requirements of eukaryotes and prokaryotes, unless the latter have an unidentified substitute “reductone”. Additionally the requirement for ascorbate has driven the evolution of at least three different biosynthetic pathways and the production of D-erythroascorbate, a 5C analogue, in fungi. Given this situation, it is interesting that the literature on ascorbate functions in mammals, and particularly in work using cultured mammalian cells, has a focus its pro-oxidant effects [3], [4], which has perhaps coloured perceptions of its function. There are likely to be differences in the handling of exogenous ascorbate by mammals with or without ascorbate biosynthesis capacity considering that primates have been evolving for 60 million years without biosynthesis [5]. Comparison between rodent models and humans is therefore not straightforward [3]. Furthermore, supplementation studies and nutritional trials have mixed results in supporting claims that large doses have specific health benefits. This review will focus on the biochemistry of ascorbate in relation to its proposed functions in plants and will make selected comparisons to mammals.

## 2 Ascorbate chemistry: antioxidant and other functions

**Fig. 1:**
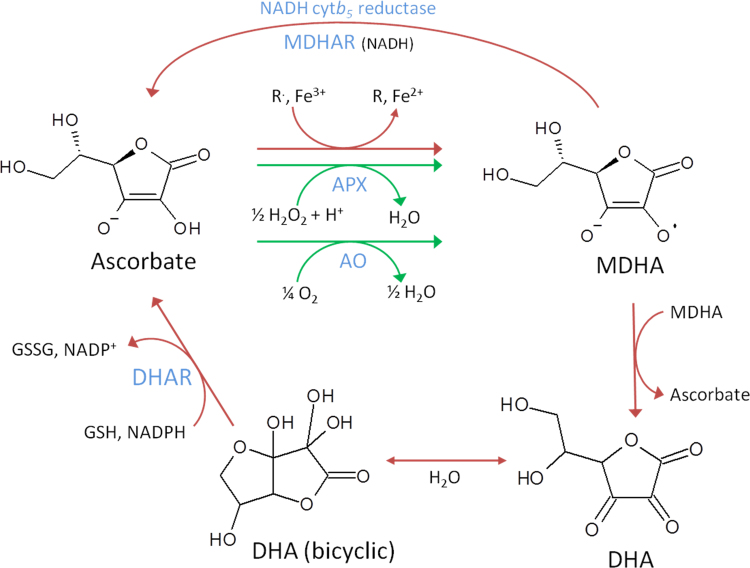
The redox reactions of ascorbate. Ascorbate is mostly present in the ionised form (Asc^-^) at pH 7.0. Its reaction with free radicals (R∙), Fe (or Cu^2+^) and H_2_O_2_ produces the resonance-stabilised MDHA radical which is central to its biological role. Reactions occurring in plants and mammals are shown with red arrows and plant-specific reactions are shown with green arrows. MDHA and DHA reduction are catalysed respectively by NADH and GSH-dependent enzymes families in plants and by a variety of enzymes in mammals.

The chemistry of ascorbate is surprisingly complex and has been well-reviewed [6], [7], [8]. The redox reactions of ascorbate are shown in Fig. 1. The one electron oxidation product of ascorbate is the monodehydroascorbate (MDHA) radical and this is probably the key determinant of its biological role. Because of resonance stabilization, MDHA does not readily react with oxygen or other molecules to generate more reactive radicals and hence is very effective as a radical scavenger. Ascorbate reacts with biologically-generated radicals such as superoxide, tocopheroxyl radicals and alkoxyl/peroxyl radicals with rate constants of > 10^5^ M^-1^ s^-1^ [9], [10]. Therefore, at sufficiently high concentration, ascorbate could complement SOD in superoxide removal *in vivo* and has the potential to regenerate tocopherol from tocopheroxyl radicals *in vivo* [11], [12]. A possible superoxide removing function was indeed pointed out by Halliwell and Foyer some time ago [13]. Superoxide reacts very rapidly with NO to produce peroxinitrite, which can then generate further radicals and cause tyrosine nitration. Ascorbate is able to effectively scavenge superoxide and prevent tyrosine nitration *in vivo* at a concentration of 10 mM [14]. While the range of intracellular ascorbate concentrations in mammalian cells is 0.1–5 mM [8] and therefore may not be significant for superoxide scavenging, it approaches 10–25 mM or more in chloroplasts [15], [16] and 10 mM in neurons [17] and could therefore cooperate with SOD to remove superoxide and decrease peroxinitrite formation. Ascorbate can reduce amino acid radicals (*e.g.* tyrosine, tryptophan) effectively [18], [19]. In this respect it is more reactive than GSH and also could be seen as a preferred route because one electron donation by GSH produces the reactive and damaging thiyl radical [18]. Conversely, ascorbate can autoxidise, generating superoxide and its dismutation product H_2_O_2_ (rate constant 3 * 10^2^ M^-1^ s^-1^). However, this reaction depends on the di-anion form which is only substantially abundant at very high pH [8]. MDHA dismutates to produce dehydroascorbate (DHA) plus ascorbate in an equilibrium reaction that is far in favour of ascorbate +DHA. While the structure DHA is often shown as a tri-carbonyl, this form is extremely unstable and in solution it is almost entirely present in the hydrated hemiacetal form (Fig. 1) [20]. In this review DHA refers to both forms.

In contrast to its relatively fast reaction with superoxide and other radicals, ascorbate reacts slowly with H_2_O_2_ (rate constant 2 M^-1^ s^-1^ at pH 7) [21]. In green plants and a number of other photosynthetic protists, H_2_O_2_ removal is catalysed by a specialised family of haem containing ascorbate peroxidases (APXs). Although APX activity has been reported in a number of non-plants, it appears that a fundamental difference between mammals and plants is an additional high capacity H_2_O_2_ ascorbate-based scavenging system [2], [22] in the latter to complement catalase as well as the more widely conserved peroxiredoxin and glutathione peroxidase-like systems [23], [24]. Plants also possess specialised enzymes for regenerating ascorbate from MDHA and DHA, which are not present in animals. However, animals do contain different enzymes with these activities (Section 8). MDHA radicals can be detected by electron paramagnetic resonance spectroscopy *in vivo* and increase in leaves under oxidative stress [25]. Ascorbate has been reported to reduce the sulfenic acid form of 1-Cys peroxiredoxin to the thiol state [26]. This is surprising and more work is needed to determine the physiological significance of this reaction.

Because of its electron donating activity, ascorbate can cause radical production and therefore it can act as a pro-oxidant. Pro-oxidant effects are most likely to occur when ascorbate concentration is low so that balancing antioxidant effects are small. A well-characterised pro-oxidant effect of ascorbate derives from its ability to reduce Fe^3+^ and Cu^2+^, resulting in hydroxyl radical production in the Fenton reaction between Fe^2+^/Cu^+^ and H_2_O_2_ [9]. Fe^2+^ can also reduce oxygen to superoxide, with generation of H_2_O_2_. If “free” redox-active metals are present then the pro-oxidant effect of ascorbate is greatly increased. This effect underlies the reports of ascorbate toxicity in mammalian cell cultures and the low (or lack of) ascorbate addition to culture media [3], [8], [27]. A critical study which varied ascorbate and Fe concentration in plasma provided no evidence for a pro-oxidant effect (lipid peroxidation) and rather showed a protective effect of ascorbate [28]. Large doses of ascorbate have been proposed as a cancer therapy because the altered metabolism of tumour cells makes them sensitive to its pro-oxidant effects [8], although ascorbate-mediated epigenetic effects have been proposed as well (Section 7). However, to achieve sufficiently high plasma concentration to damage tumour cells, it must be delivered intravenously to bypass the normal control of plasma ascorbate levels by uptake by the gut. Non-enzymatic DHA degradation also generates H_2_O_2_ and might provide a significant H_2_O_2_ source in plant cell walls [29]. The Fe reducing ability of ascorbate is the basis of its best-known function in maintaining the activity of 2-oxoglutarate-dependent dioxygenases (Section 7).

The ability of ascorbate to reduce Fe^3+^ suggests that it could have a physiologically significant role in Fe uptake. Plants have two strategies for extracellular Fe uptake. Fe^3+^ reduction by ferric chelate reductase (FRO, a plasma membrane enzyme related to NAPDPH oxidase) followed by Fe^2+^ uptake *via* an iron transporter, IRT (Strategy I). This system is induced by iron deficiency and also involves enhanced extracellular acidification by the H^+^-ATPase and citric acid efflux. In contrast, some monocotyledonous plants secrete a siderophore which chelates Fe^3+^ and the complex is absorbed (Strategy II) thus avoiding extracellular reduction [30]. Recent evidence suggests that ascorbate efflux additionally plays a role in Fe^3+^ reduction in pea and Arabidopsis (Strategy I plants) and facilitates Fe uptake into developing embryos. FRO activity is low in embryos but ascorbate secreted into the apoplast (the cell wall/extracellular space of plants) reduces extracellular Fe^3+^. This mechanism is supported by genetic evidence, since the embryos of ascorbate deficient *vtc2–4* and *vtc5* Arabidopsis mutants have decreased Fe^3+^ reducing capacity and a 75% decrease in seed Fe concentration [31]. The presence of ascorbate in the apoplast is well characterised, along with an apoplastic enzyme ascorbate oxidase, which affects its oxidation state [32]. However, critically, the nature of the transporters which enable ascorbate efflux and dehydroascorbate uptake in plants is unknown. Fe deficiency in the green alga *Chlamydomonas* causes a very large increase in ascorbate and expression of the homologue of the Arabidopsis biosynthesis gene *VTC2*, suggesting a role in iron uptake [33]. Ascorbate may also play a role in facilitating Fe uptake and homeostasis in mammals [34], [35].

As well as redox-related reactions, ascorbate reacts with electrophiles and DHA reacts with nucleophiles. Cosequently, a number of naturally-occurring conjugates have been reported [36], [37], [38], [39]. Ascorbate is involved in the specialised glucosinolate (mustard oil glycoside)-based defence mechanism of cruciferous plants. During attack by insects, glucosinolates are broken down to release isothiocyanates or nitriles, the former acting as a feeding deterrent for generalist herbivores or as an attractant for specialized herbivores [40]. Ascorbate and indole-3-carbinol, a breakdown product of indole glucosinolates, react to form ascorbigen [36], [41]. Glucosinolates are hydrolysed by myrosinase, a thioglucosidase which has an ascorbate requirement. Uniquely, ascorbate is associated with the active site of myrosinase and is part of its catalytic mechanism [42].

DHA reacts with cysteine, lysine and arginine residues to form dehydroascorbylated proteins [43], [44], [45], [46], [47] and also reacts with guanosine [48]. In the case of the eye, which has high ascorbate concentration, DHA and its breakdown products cause glycation of eye lens proteins such as crystallin [49] and a GSH-DHA adduct forms in Jurkat cells fed with DHA [50]. It is possible that dehydroascorbylation could act as a specific regulatory post-translational protein modification responding to increased ascorbate oxidation rather than as generic damage, but so far there is no evidence for this role.

## 3 Naturally-occurring ascorbate derivatives and analogues

**Fig. 2:**
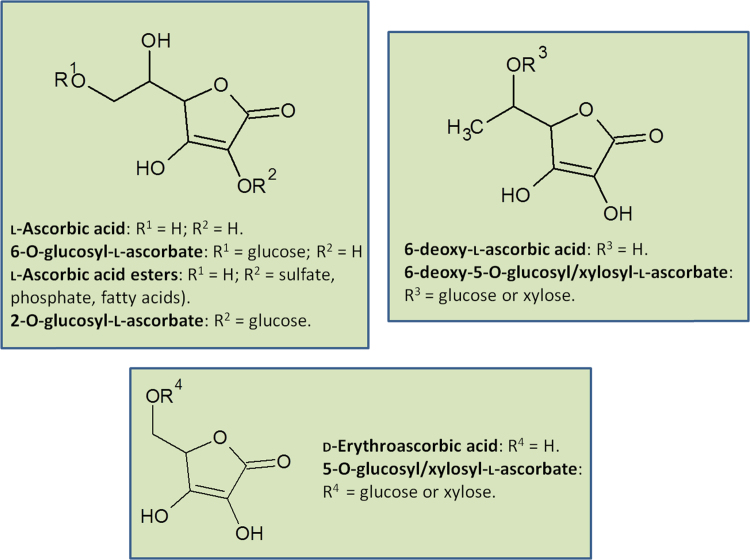
The diversity of ascorbate and its derivatives. All are naturally occurring with the possible exception of the phosphate and fatty acid esters. However, the esterification of ascorbate at C2 and 2-O-glycosylation protects the reactive *ene*diol group allowing these derivatives to be used as stable food supplements.

A range of ascorbate-like compounds occur in different groups of organisms and there is diversity in the occurrence of esters and glycosides (Fig. 2). Ascorbate is the most widely distributed and is the only form present in mammals. However, brine shrimp cysts contain ascorbate 2-sulfate which is hydrolysed to release ascorbate when the shrimps become active [51], [52]. Derivatisation of the 2-position stabilises ascorbate against oxidation. Two ascorbate derivatives have been identified in plants: 6-O-glucosyl in curcurbits (squashes) [53] and 2-O-glucosyl in goji berry (*Lycium barbarum*) fruit [54]. Fungi diverge from other organisms in synthesising D-erythroascorbate, a C5 analogue along with its 5-O-glycosides [55], [56], [57], [58], [59]. Basidiomycete fungi additionally make 6-deoxyascorbate and its 5-O-glycosides [60]. Ascorbate 6-phosphate is synthesised by *E. coli* as part of its ascorbate uptake and utilisation pathway [61]. A systematic survey of the under-explored groups of eukaryotes might reveal further diversity of ascorbate analogues (reductones) or replacements that satisfy the key features of an *ene*diol group which acts as a one electron donor antioxidant forming a “resonance stabilised” free radical oxidation product.

## 4 Ascorbate biosynthesis

**Fig. 3:**
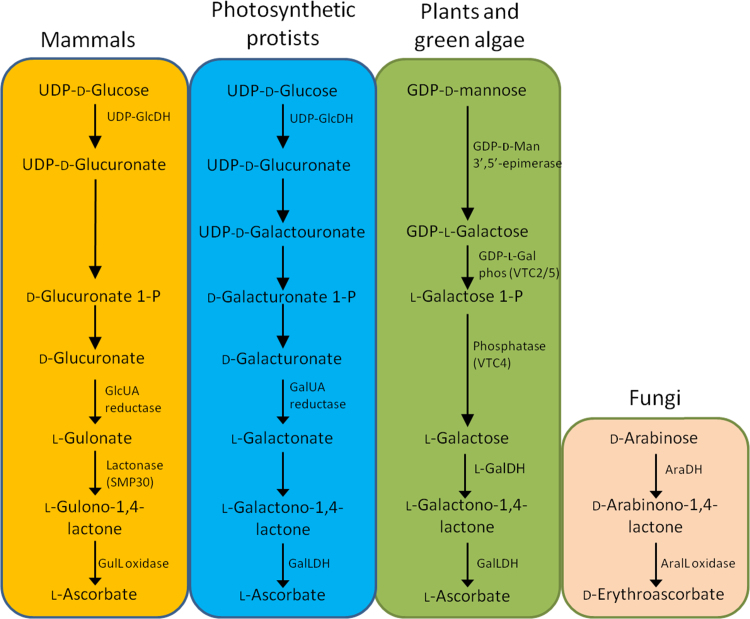
The currently known ascorbate biosynthesis pathways in mammals, plants, photosynthetic protists and fungi. In all cases, the final step is oxidation of an aldono-1,4-lactone to ascorbate using an FAD-linked oxidase or dehydrogenase. The oxidase generates H_2_O_2_ as well as ascorbate. The series of reactions generating the aldono-1,4-lactone differs between groups. Photosynthetic protists evolved from a secondary endosymbiosis between a non-photosynthetic ancestor and algae, so it is proposed that this pathway represents a hybrid of the plant and mammalian pathways [2].

Ascorbate is most likely not made or required by prokaryotes. Selected eukaryote groups (including primates and teleost fish) have lost a functional gene encoding the last enzyme in the pathway and have to obtain dietary vitamin C [1]. Analysis of genome sequences suggests a few other eukaryotic groups (*e.g.* ciliates) may also lack biosynthetic capacity [2]. Ascorbate biosynthesis pathways differ between animals, green plants and photosynthetic protists [2], [62] while, as noted in the previous section, fungi synthesise a 5C analogue of ascorbate, D-erythroascorbate. Recent evidence suggests that the nematode *Caenorhabditis elegans* contains ascorbate but does not use any of the known pathways [63]. Ascorbate biosynthesis pathways have been well-reviewed [2], [57], [64], [65], [66] and recent developments are minimal, so they will only be outlined here (Fig. 3). In green plants, despite repetitive claims in numerous publications, there is only definitive evidence for the D-mannose/L-galactose (Smirnoff-Wheeler) pathway but tantalising suggestions for incorporation of D-galacturonic acid in some tissues such as tomato fruit [67]. In mammals, ascorbate is synthesised in the liver but, presumably because it is not made by humans, details of pathway control have received very little attention since the 1960s. Knockout mutants in mice [68] have identified the lactonase (Fig. 3) and provide experimental material for probing ascorbate function more deeply. The final step in the mammalian pathway is catalysed by L-gulono-1,4-lactone oxidase, an FAD-linked enzyme which appears to be associated with the endoplasmic reticulum (Fig. 5). Its precise location is not known but it is assumed to produce ascorbate on the lumenal side of the ER membrane with the production of H_2_O_2_ [64]. A number of generally un-testable hypotheses have been advanced to explain loss of ascorbate biosynthesis capacity in primates, teleost fish and other scattered animal species, including avoidance of H_2_O_2_ production by gulonolactone oxidase and replacement of ascorbate with uric acid as an antioxidant since the loss of gulonolactone oxidase is correlated with loss of uricase [69]. The UDP-glucuronate used for ascorbate synthesis is also required for detoxification and excretion of toxins and drugs by glucuronidation. It is possible that removal of ascorbate biosynthesis, which is estimated to comprise 30% of the flux through UDP-glucuronate in rat liver [64] improves the capacity to detoxify dietary phytochemicals by glucuronidation. Beyond this, any other factors controlling the rate of ascorbate synthesis in mammals are not known. In plants and green algae, the current view is that control of pathway flux resides largely at the GDP-L-galactose phosphorylase step which is encoded by the paralogues *VTC2* and *VTC5* in *Arabidopsis thaliana* [65], [70]. This is the first step in the D-mannose/L-galactose pathway that is dedicated to ascorbate synthesis. Arabidopsis leaf ascorbate concentration adjusts to light intensity over a period of 4 days [71] showing a close relationship with the photosynthetic light response curve. *VTC2* and *VTC5* transcripts are increased by high light intensity, possibly *via* a photosynthesis-sourced signal [72], [73], [74]. Ascorbate accumulation is induced by low and high temperature and nitrogen deficiency [75], [76]; stresses which limit photosynthesis rate and, like high light intensity, give rise to excess excitation energy. *VTC2* and *VTC5* expression does not respond to H_2_O_2_ or other oxidative stresses suggesting that the signal is not ROS related, although algae differ since the *Chlamydomonas VTC2* orthologue is strongly induced by H_2_O_2_, singlet oxygen and *tert*-butyl hydroperoxide [77], [78], [79]. To maintain the appropriate ascorbate concentration, its rate of synthesis is repressed as ascorbate increases and the rate of breakdown increases [80]. *VTC2* is proposed as the step responsible for feedback inhibition *via* decreased translation. Laing *et al*. suggest that a small ascorbate binding peptide, encoded by a uORF, influences translation of VTC2 mRNA. This is an intriguing hypothesis but the existence of this peptide and its effect on translation have not been directly measured. The last step of ascorbate biosynthesis in plants is catalysed by L-galactono-1,4-lactone dehydrogenase. It is located in complex 1 of the mitochondrial electron transport chain (Fig. 3, Fig. 5) and, as well as forming ascorbate [81], [82], [83], it is required as a chaperone for assembly of complex 1 [84], [85], [86]. This dual role is intriguing and suggests a link between mitochondrial function and ascorbate whose role is not entirely obvious.

## 5 Ascorbate and dehydroascorbate catabolism

**Fig. 4:**
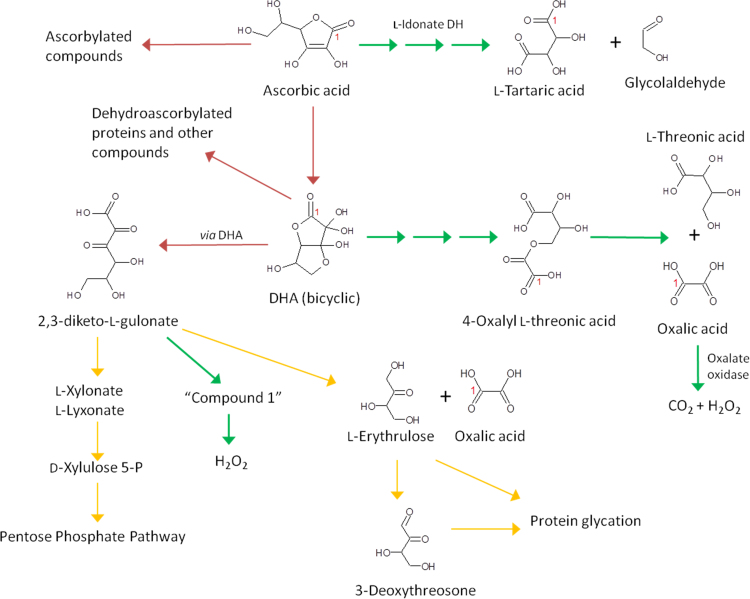
Ascorbate degradation pathways. Ascorbate is enzymatically converted to tartaric acid in some plants (*e.g.* grapes). DHA undergoes a complex set of reactions, some involving unidentified enzymes, many of which occur in extracellular fluid. There is evidence that oxalate production could also be intracellular in plants. Some of the products are reactive and potentially damaging carbonyl compounds. General pathways are shown with red arrows, plant-specific pathways with green arrows and those demonstrated in mammals with yellow arrows.

The ascorbate pool undergoes turnover in plants [80], [87], [88], [89]. Turnover is increased by oxidative stress thus decreasing the total ascorbate pool. For example, this occurs in catalase mutants and when catalase activity is inhibited by aminotriazole [90], [91]. Ascorbate catabolism causes extensive post-harvest loss from salad leaves [92]. The additional radical production caused by smoking results in a increased daily requirement for ascorbate to maintain plasma concentration [93]. In leaves, there is a strong relationship between ascorbate and light *via* light-induced and circadian expression of the biosynthetic genes *VTC2* and *VTC5* (Section 4). Through normal light/dark cycles ascorbate only fluctuates to a small extent but when the dark period is extended, ascorbate pool size and *VTC2/5* expression are decreased [72], [87], [88], [89]. The rapid ascorbate loss in extended dark occurs along with the induction of a range of carbohydrate starvation responses that are induced by this treatment [94]. The final stable products of DHA catabolism differ between species, with oxalic and threonic acid being the most common [89], [92]. The pathway for production of these compounds from DHA under apoplastic conditions involves a mixture of non-enzymatic and enzymatic steps including intermediates such as 4-O-oxalyl L-threonate (Fig. 4) [29], [95]. DHA also hydrolyses to 2,3-L-diketogulonate. This compound, under apoplastic conditions, is oxidised to a currently unidentified compound (Compound 1) which can generate H_2_O_2_ non-enzymatically and also inhibits peroxidase. Interestingly, compound 1 is destroyed by ascorbate oxidase (a prominent cell wall localised enzyme) so may have an *ene*diol group [96]. These reactions provide the possibility that this DHA derivative could influence pathogen defence responses and cell wall polymer cross linking processes that depend on H_2_O_2_ and type III peroxidases. Studies of ascorbate/DHA breakdown products have to a large extent been carried out under apoplastic conditions and with exogenously-supplied ^14^C-ascorbate. The extent to which ascorbate or DHA are degraded by similar intracellular pathways is unknown. A number of plant species accumulate calcium oxalate crystals in specialised crystal idioblast cells. Labelling and genetic evidence suggests that the oxalate is derived from ascorbate [97], [98], [99]. The pathway or its cellular location is not known although *Medicago truncatula* mutants with increased oxalate may provide information [99]. Oxalate can also be synthesised from glyoxylate or oxaloactetate and, in rice, it is probably derived from glyoxylate [100]. L-Tartaric acid is a product of ascorbate in plants belonging to the families Vitaceae and Geraniaceae. Red wine drinkers will be familiar with the crystalline precipitate that contains insoluble tartaric acid salts derived from grape ascorbate. In contrast to threonate/oxalate production, this 5 step pathway in grapes uses ascorbate not DHA as precursor. One enzyme, L-idonate dehydrogenase, has been identified [101], [102]. In mammals, as in plants, DHA gives rise to diketogulonate, threonate and oxalate. Additionally, in the eye ascorbate-derived L-erythrulose gives rise 3-deoxythreosone, a reactive osone that glycates lens proteins [103], [104] and C5 aldonic acids which may be converted to D-xylulose 5-P and enter central metabolism *via* the pentose phosphate pathway [64], [105], [106]. The formation of kidney stones composed of calcium oxalate is often cited as a potential danger of ingesting large amounts of ascorbate but this is probably unlikely in healthy people [3]. In summary, DHA has a complex chemistry and *in vivo* can produce a range of products some of which are reactive and can damage proteins or give rise to H_2_O_2_. Consequently the ascorbate pool must be maintained by continual biosynthesis or dietary intake, the requirement increasing when DHA production increases during oxidative stress.

## 6 Ascorbate distribution and transport

**Fig. 5:**
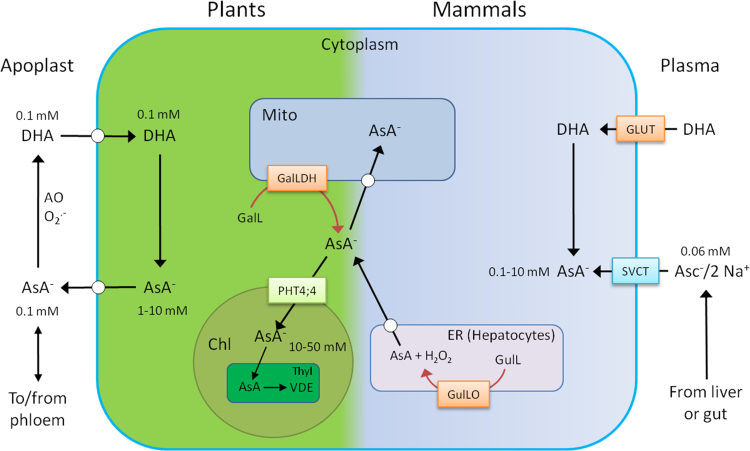
A comparison of the sites of ascorbate synthesis and transport in plants and mammals. Ascorbate (SVCT) and DHA (GLUT) transporters allow mammalian cells to accumulate ascorbate from low plasma concentrations. SVCTs are co-transporters that use a transmembrane Na^+^ gradient to drive uptake while GLUTs allow facilitated transport of DHA, which is reduced inside the cell by DHAR enzymes. In contrast, uptake by plant cells may be primarily *via* DHA although the molecular nature of transporter is unknown. There is also an ascorbate efflux mechanism, possibly *via* an anion channel. An ascorbate transporter allowing movement into the chloroplasts (PHT4:4) has been identified recently. Also, in plants apoplastic ascorbate oxidase maintains the ascorbate pool in a more oxidised state than the intracellular pool. Peroxisomes and vacuoles are not shown in this scheme but both contain ascorbate. AsA^-^, ascorbate; AO ascorbate oxidase; Chl, chloroplast; DHA dehydroascorbate (bicyclic); ER, endoplasmic reticulum; GalL, L-galactonolactone; GalLDH, L-galactonolactone dehydrogenase; GLUT, DHA transporter; GulL, L-gulonolactone; GulLO, L-gulonolactone oxidase; Mito, mitochondrion; PHT4;4, ascorbate transporter; SVCT, ascorbate-Na co-transporter; Thyl, thylakoid; VDE, ascorbate-dependent violaxanthin de-epoxidase. Shaded rectangles, enzymes and transporters; white circles; transport activities using unidentified proteins.

Ascorbate concentration in plants varies between tissues, with highest concentrations in leaves and flowers and lower concentrations in less photosynthetically active tissues such as stems and roots [32]. Ascorbate and DHA disappear from developing seeds as they mature and dehydrate and is accumulated during imbibition and early germination [107], [108], [109]. Ascorbate occurs in all compartments of plant cells in mM concentrations and in the extracellular space (apoplast) where its concentration is 5–10 times lower and it is more oxidised than the intracellular pool (Fig. 5). In Arabidopsis, the typical leaf ascorbate concentration (2–10 µmol g^-1^ fresh weight) is higher than any other primary metabolite [110] Ascorbate is transported long distance in the phloem so can move from photosynthetically active leaves to sink tissues such as roots [111], [112]. The mechanism of ascorbate transport in plant cells is a major gap in knowledge. A high affinity uptake system (K_m_ 40 µM) has been demonstrated in Arabidopsis cell cultures which is specific to DHA. Using careful control of ascorbate oxidation to DHA, it was shown that ascorbate transport is negligible [113] and previous reports of ascorbate uptake [114], [115] are likely to be due to oxidation of supplied ascorbate. Plant cell cultures rapidly oxidase ascorbate and this is most likely mediated by the apoplastic ascorbate oxidase (Section 8). Cultured cells exposed to H_2_O_2_ release a pulse of ascorbate, which oxidises in the culture medium and is then re-absorbed indicating a possibility that oxidative stress activates an ascorbate efflux system to protect the apoplast [116]. Recently, electrophysiological measurements have provided evidence for ascorbate efflux *via* an anion channel which is activated by gluconic acid and blocked by anthracene-9-carboxylic acid [117]. The transport proteins responsible for DHA uptake and ascorbate efflux have not yet been identified. Ascorbate/DHA uptake by isolated vacuoles or thylakoid membranes is non-saturable, so may not involve a specific carrier [114], [115]. Ascorbate uptake by chloroplasts is carrier mediated but it is only recently that a transporter was identified as PHT4;4, a member of a phosphate transporter family. Mutants of this transporter had decreased chloroplast ascorbate concentration and impaired non photochemical quenching (an ascorbate-dependent process- Section 7) [118], [119]. Considering the amount of residual ascorbate in the chloroplasts of this mutant, other transporters may be required. Mitochondria from tobacco BY2 cells take up ascorbate and DHA (K_m_ 36 µM and 6 µM respectively, with similar V_max_ for both). Uptake of DHA was preferred and, based on inhibitors and competition experiments, DHA uptake could use, or share the use of a glucose transporter [120]. However, since the intracellular ascorbate pool is usually > 90% reduced, it is likely that mitochondrial uptake involves ascorbate as well as DHA.

In mammals, ascorbate uptake across the plasma membrane is mediated by Na-dependent transporters (SVCTs). This process allows concentration from the micromolar plasma concentrations to intracellular concentrations in the millimolar range [8], [121]. As a broad generalisation, the highest concentrations occur in neurons/brain, the eye, phagocytes and the adrenal gland which are tissues particularly exposed oxidative stress or, in the case of the adrenal gland, which house hormone synthesising 2-oxoglutarate-dependent dioxygenases. The occurrence of very high ascorbate in neurons seems to be related the very high aerobic respiration rate which could lead to superoxide production in mitochondria but additional roles in neurotransmission and brain metabolism are evident [122], [123], [124], [125], [126], [127]. DHA uptake is facilitated by GLUTs (glucose transporters), so it follows concentration gradients, allowing reduction to ascorbate in the cell (by DHAR type enzymes) to drive uptake. In humans, there is genetic variation in SVCT expression and it can also decline with age [128]. Therefore, the extent of dietary intake to satisfy demand will vary between individuals. Modulation of transport activity is potentially a way to control intracellular ascorbate status in mammalian cells since, even in those species able to synthesise it, it is distributed from its main site of synthesis in the liver. Ascorbate is often present at low concentration in mammalian cell culture media [129]. This state of affairs seems to be driven by the artefactual pro-oxidant effect of ascorbate in culture media [130], [131]. Therefore, there is a possibility is that many studies with cell cultures (even in ascorbate synthesising species) will be based on abnormally ascorbate deficient cells, resulting in lack of understanding of its functions or translation to whole bodies [3]. This is being highlighted by recent advances in its effect on epigenetic control of gene expression *via* 2-ODDs (Section 7).

## 7 Ascorbate-dependent enzymes

A number of enzymes require ascorbate and it has distinct roles as a “chaperone” to maintain active centre Fe^2+^ (2-oxoglutarate-dependent dioxygenases), a catalytic role in the active site of myrosinase (Section 2) and as a substrate (violaxanthin de-epoxidase). Other enzymes could be affected by ascorbate, an example being mitochondrial glycerol 3-phosphate dehydrogenase from guinea pigs. It is activated several-fold by ascorbate and is proposed to be involved in ascorbate-stimulated insulin release [132].

### 7.1 2-oxoglutarate-dependent dioxygenases: a renaissance in attention for ascorbate

The archetypal understanding of ascorbate function derives from the severe deficiency disease scurvy, which is now rare. The various symptoms are consistent with a lack of collagen, a protein critical for the structure of the extracellular matrix. Collagen contains hydroxyproline and hydroxylysine residues essential for its structural properties and these are formed post-translationally by peptidyl prolyl hydroxylase [133]. This enzyme is a 2-oxoglutarate dependent dioxygenase (2-ODD) with Fe in its active site. Ascorbate is an effective chaperone for this enzyme because, if substrate does not bind, the enzyme undergoes an uncoupled reaction cycle resulting in the active site Fe becoming stalled in a high oxidation state. Ascorbate reduces it back to Fe^2+^ and restores activity [134], [135]. Other 2-ODDs synthesise carnitine and adrenaline/catecholamines which impact energy metabolism during the development of scurvy [8]. While ascorbate is the normal reductant and cannot be replaced [136], [137] there are some 2-ODDs in which glutathione can maintain activity [138]. Considering that 2-ODDs are present in prokaryotes that lack ascorbate, the enzymes may vary in likelihood of uncoupled reaction cycles occurring as well as ability to use a thiol reductant [137]. Presumably one electron reduction of Fe by GSH would produce a potentially damaging thiyl radical. 2-ODDs are involved in hydroxylation of HIF (hypoxia inducible factor). This provides an oxygen sensing mechanism in which high oxygen concentration increases hydroxylation of prolyl residues leading to ubiquitination and proteolytic degradation of HIF [137]. As noted above there seems to be variation between HIFs in the ability of GSH to replace ascorbate [137], [138].

Recently, in mammals an interest in ascorbate has received a boost by the recognition that 2-ODDs are involved in histone and DNA demethylation and therefore in the epigenetic regulation of gene expression [129]. TET (ten eleven translocation) proteins are 2-ODDs that convert 5-methylcytosine (5mC) to 5-hydroxy-methylcytosine (5hmC), 5-formylcytosine (5fC) and then 5-carboxylcytosine (5caC). 5fC and 5caC are then replaced by cytosine by base excision repair machinery [139]. Ascorbate, but not other antioxidants, increases TET-dependent 5hmC production and cytosine demethylation in mice embryonic stem cells leading to changes in development [136]. TET2 is involved in differentiation of haematopoietic stem cells (HSCs). In humans and L-gulonolactone oxidase mutant mice studies, ascorbate promoted TET2-dependent 5hmC formation allowing normal differentiation while ascorbate or TET2 deficiency allows HSCs to proliferate resulting in leukaemia [140], [141], [142]. Histone demethylation requires Jumonji C-domain-containing histone demethylases, which are also ascorbate-dependent 2-ODDs, thereby providing a further means for ascorbate to influence gene expression [129], [139]. The possibility of ascorbate influencing cell differentiation and therefore cancer raises the question of the extent to which it might have a regulatory influence. The observation that HSCs contain very high ascorbate [141] raises the possibility that accumulation in specific cell types *via* high SVCT expression could control TET activity and differentiation. It also highlights the importance of ascorbate status in cell culture studies [129].

Plants have numerous 2-ODDs involved in hormone synthesis (ethylene, abscisic acid, gibberellins) and degradation (IAA) and synthesis of a wide range of secondary compounds, including anthocyanins and glucosinolates [143], [144], [145]. Also, cysteine oxidase oxidises N terminal cysteines to mark proteins for degradation *via* the N-end rule [146]. Similarly to animals, plants have hydroxyproline containing extracellular matrix proteins such as extensin and type III peroxidases [147]. The question therefore arises if ascorbate deficient mutants suffer from scurvy-like symptoms. Measurement of hydroxyproline in hydrolysed cell wall pellets of wild type and *vtc1* and *vtc2* ascorbate deficient Arabidopsis mutants found no differences showing that decreasing ascorbate to 20% of normal does not affect proline hydroxylation [148]. However, ascorbate deficient mutants are impaired in high light-induced anthocyanin accumulation, a process requiring several 2-ODDs but this seems to be caused by impaired induction of anthocyanin biosynthesis gene expression suggesting an indirect effect of ascorbate on high light signalling [71] possibly through an influence on H_2_O_2_, since anthocyanin accumulation is also suppressed in a catalase mutant [149].

### 7.2 Violaxanthin de-epoxidase (VDE)

Violaxanthin de-epoxidase (VDE) is a plant-specific enzyme in the lipocalin family which uses ascorbate as reductant in the de-epoxidation of the xanthophyll pigment violaxanthin to produce zeaxanthin [150]. The enzyme is localised on the lumen side of the thylakoid membranes in chloroplasts and is activated by the decrease in lumenal pH that results from photosynthetic electron transport. Zeaxanthin is involved in the process of non photochemical quenching (NPQ) which is essential for protecting photosynthesis from damage by intense light [151]. Excess excitation energy in the PSII light harvesting complexes is transferred to zeaxanthin, which is then able to dissipate this energy harmlessly as heat. Arabidopsis ascorbate-deficient *vtc* mutants are impaired in the speed and extent of NPQ development and zeaxanthin accumulation in high light and are more sensitive to photooxidative stress [152], [153], [154], [155]. As noted by Foyer and Lelandais [114], the apparent lack of a transporter to move ascorbate into the thylakoid lumen suggests that a high ascorbate chloroplast stroma concentration is needed to maintain VDE activity. The possibility that a protein from a phosphate transporter family (PHT4;1) could be a thylakoid membrane ascorbate transporter requires investigation [118].

## 8 Ascorbate oxidising and recycling enzymes

### 8.1 Ascorbate peroxidase (APX)

Ascorbate reacts with hydrogen peroxide with a rate constant of 2 M^-1^ s^-1^ [21] so the reaction will be slow under physiological conditions. Therefore, efficient H_2_O_2_ removal by ascorbate requires catalysis and, while APX activity has been reported in a range of species, a haem peroxidase with high specificity for ascorbate is largely confined to green plants and photosynthetic protists [2]. For this reason ascorbate is not seen as a key player in H_2_O_2_ removal in mammals. Plant APX has been very well reviewed recently [156], so only a few points will be summarised here. Around eight APX genes occur in Arabidopsis, some being targeted to more than one location. In chloroplasts, there are two APXs, on in the stroma and the other associated with the thylakoid membrane near PSI. They are both sensitive to inactivation by H_2_O_2_ compared to cytosolic isoforms, particularly if ascorbate concentration is low. This sensitivity is decreased by mutation of specific amino acid residues [157], [158]. The authors suggest that engineering plants with H_2_O_2_ resistant chloroplast APXs could improve stress resistance, however an alternative view is that H_2_O_2_ sensitivity enables H_2_O_2_ to escape the chloroplast and act as a signal [159], [160]. APX is activated by nitrosylation and its activity is also affected by nitration and carbonylation, providing a potential for levels of RS to influence H_2_O_2_ scavenging capacity [161], [162], [163]. Numerous studies with mutants and APX over-expressing plants have confirmed the important role of APX in protecting plants from oxidative stresses and have revealed reams of genes whose expression is changed [164]. Because there is a parallel set of multiple thiol peroxidase enzymes (peroxiredoxins and glutathione peroxidase-like), plants with multiple mutations are needed to assess their interacting functions [23], [24]. The expression of various APX genes is affected by environmental conditions and it is noteworthy that the Arabidopsis cytosolic forms (APX1 and APX2) are very responsive to high light and H_2_O_2_ [156], [165], [166], [167].

### 8.2 Dehydroascorbate reductase (DHAR)

Glutathione reduces DHA slowly under physiological conditions but the reaction is catalysed by a wide range of enzymes in plants and mammals. These include the plant glutathione-dependent DHARs (which are part of the glutathione S-transferase superfamily), glutaredoxin, thioredoxin (NADPH-dependent) and protein disulfide isomerase [168], [169], [170], [171], [172], [173]. Plants have multiple isoforms of GSH-dependent DHARs which are targeted to cytosol, chloroplasts, mitochondria and peroxisomes with K_m_ values for DHA and GSH in the 0.1 and 5 mM range respectively [169], [172]. Crystal structures and reaction mechanisms have been proposed for the green plant enzyme [174], [175], [176]. Both suggest that an active site cysteine is converted to the sulfenic acid form due to oxidation by DHA, which itself is reduced to ascorbate. This reaction also requires input of one water molecule. Both studies show the substrate as the (very unstable) tri-carbonyl form of DHA, but it is generally accepted that it hydrates in solution to the bicylic hemiacetal form [6] and possibly the hydrated substrate supplies the necessary oxygen for sulfenic acid production. In this reaction mechanism, DHA is not covalently bound to the enzyme but, in liver glutaredoxin which reduces DHA to ascorbate with similar K_m_ values to the plant DHARs, DHA is proposed to be covalently bound to the active site cysteine as a thiohemiketal intermediate [171]. Knockout mutants of Arabidopsis DHAR1 (cytosolic and peroxisomal), and 2 (cytosolic) and DHAR3 (chloroplastic) have little difference in total ascorbate (ascorbate + DHA) concentration until exposure to high light intensity. The GSH pool is less oxidised in the DHAR3 mutant (one allele only) under low or high light but only under high light in DHAR1 mutants, perhaps reflecting a greater rate of ascorbate oxidation in chloroplasts compared to cytosol in low light [177], [178]. In low light the oxidation state of the ascorbate pool (DHA/total ascorbate) is unaffected in DHAR mutants and is around 0.9, a value typical of a wide range of studies. Rather inexplicably this value decreases to 0.5 in HL treated plants in one study but not the other, but in either case wild type and mutants do not have a different ascorbate oxidation state. Along with greater loss of ascorbate in mutants during HL stress, this observation would suggest that DHA that is not reduced is degraded and lost to the ascorbate pool. In leaves, large proportions of DHA are probably only present under extreme conditions and furthermore, close examination of the literature suggests that DHA/total ascorbate increases as total ascorbate decreases, suggesting this ratio mostly represents a constant DHA concentration, possibly produced during extraction. The Arabidopsis DHAR1 and 3 mutants do clearly show that DHA recycling is required for tolerance to photo-oxidative stress. Studies with various plant species show increased ascorbate pool size, sometimes increased GSH, and improved tolerance to oxidative and related stresses (at least under laboratory conditions) when DHAR is over-expressed [179], [180], [181], [182], [183], [184]. Rice grain yield was increased by ~ 15% under paddy field conditions in two seasons by over-expressing a rice DHAR [185] Stomatal guard cell closure is partly controlled by H_2_O_2_ and DHAR over-expressing plants have more open stomata, suggesting improved H_2_O_2_ removal is enabled [186]. Expression of DHAR in mammalian cells also influences ascorbate and oxidative stress tolerance [170].

### 8.3 Monodehydroascorbate reductase (MDHAR)

MDHA is the initial product of the reducing activity of ascorbate and also exists as part of the equilibrium between ascorbate and DHA (Section 2). Its presence *in vivo* is confirmed by electron paramagnetic resonance spectroscopy which shows that it increases under stressful conditions [25], [187]. Plant MDHAR is a FAD-linked reductase and has a high affinity for MDHA, which it reduces to ascorbate, and has a strong preference for NADH over NADPH [188]. A recent crystal structure of rice MDHAR provides a structural basis for this preference [189]. Although MDHAR activity has been reported in animals, no specific proteins are characterised [190] and, with a few exceptions (*e.g.* choanoflagellates) the gene family is limited to green plants [2]. MDHAR is encoded by 5 genes in Arabidopsis, with predicted targeting to cytosol, chloroplasts, peroxisomes and mitochondria. Various forms are soluble or anchored to peroxisomal [191] and plasma membranes [168]. Although MDHAR activity is reported in the apoplast of barley [192], none of the Arabidopsis proteins have predicted secretory sequences. While current reports on the consequences of manipulating MDHAR activity focus on ascorbate, there is evidence that it also reduces other radicals, for example phenoxyl radicals produced during oxidation of quercetin, ferulic acid, coniferyl alcohol and chlorogenic acid [193]. Recently, in an interesting twist, the toxicity of the explosive 2,4,6-trinitrotoluene (TNT) was shown to be dependent on mitochondrial MDHAR activity. Mutants in Arabidopsis MDHAR6 are more tolerant to TNT because they avoid reducing it to a radical which then autoxidises, generating superoxide [194]. Therefore, it is clear that MDHAR has wide substrate specificity and could be multifunctional, a possibility that should be considered when interpreting the results of over-expression experiments. Furthermore isoforms in different compartments could have different substrate preferences, as evidence by the specificity of MDHAR6 in TNT toxicity.

### 8.4 Ascorbate oxidase (AO)

Ascorbate oxidase (AO) is a blue copper oxidase which is glycosylated and secreted into the cell wall. It oxidises ascorbate with the production of water (Fig. 5) and also oxidises “Compound 1” (Fig. 4), an unidentified breakdown product of DHA [195]. Consistent with its localisation in the apoplast, mutants with decreased AO activity have a more reduced apoplastic ascorbate pool while AO over-expression in tobacco oxidises apoplastic ascorbate with no effect on intracellular ascorbate oxidation state [196], [197], [198], [199]. Apoplastic ascorbate is involved in defence against ozone in some species and tobacco AO over-expressers are more susceptible [196]. It tends to have its highest activity in areas of cell expansion and there has been speculation that it influences cell expansion and hormone and redox signalling in the apoplast [200], [201], [202], [203]. Mutants and over-expression of the enzyme, however, have not provided a clear picture of its function. It is important to note that an Arabidopsis T-DNA insertion mutants and tobacco antisense lines with less than 20% of wild type AO activity exhibit subtle changes in growth or development [197], [199]. Therefore, either AO does not have a fundamental role in plant growth or this function can be supported by 20% of normal activity. Lowered AO however does manifest noticeable effects during stress treatments (for example ozone, salt, drought and pathogen challenge) so it may be important for plant growth in natural environments [199], [204].

## 9 What have plant mutants in ascorbate metabolism revealed about its function?

In plants, mutants affected in ascorbate biosynthesis (*e.g. vtc* mutants in Arabidopsis), APX, MDHAR and DHAR have been characterised. Differences in the properties of biosynthesis and APX mutants (Section 8) could provide a tool to differentiate the functions of ascorbate itself (*e.g.* free radical removal, Fe nutrition, 2-ODDs) from its role in H_2_O_2_ removal. The most useful biosynthesis mutants will be in the first dedicated step of the pathway (GDP-L-Gal phosphorylase) because they are least effected by disruption of cell wall metabolism and protein glycosylation. In Arabidopsis, GDP-L-Gal phosphorylase is encoded by two genes (*VTC2* and *VTC5*) as described in the section on biosynthesis. The double *vtc2 vtc5* mutant cannot grow after initial germination unless supplemented with ascorbate or its immediate precursors [205], [206], showing that ascorbate is absolutely essential for plants. The challenge is to devise a strategy to identify which critical processes have failed in the double mutant: this has not yet been achieved. A simple explanation in relation to seed germination would be the large amount of H_2_O_2_ generated by fatty acid β-oxidation for powering early growth of this oilseed. This is supported by the deleterious effect of a peroxisomal MDHAR mutant [191]. However, this effect is alleviated by growth on sucrose (which suppresses fatty acid utilisation) and the double mutation is still lethal on sucrose supplemented media. *vtc2* mutants with ~ 20% of wild type ascorbate grow nearly as well as WT [206] although the widely-used *vtc2-1* allele is small [205], [207]. Its small stature can be separated from ascorbate deficiency by backcrossing suggesting that other mutations are responsible [206]. Since ascorbate is generally the most abundant primary metabolite in Arabidopsis leaves, the question of why its concentration can be decreased with such a small effect arises. The most likely explanation is that somewhere between 0% and 20% is needed to maintain basic functions so that larger amounts are not needed under benign laboratory conditions. When laboratory-grown plants are challenged by more stressful conditions, then *vtc* mutants start to display symptoms. They are more sensitive to high light, temperature extremes, salinity, ozone and a number of other stresses, particularly leading to photo-oxidative stress and, in extreme cases, cell death [75], [155], [208], [209], [210], [211]. Overall, it is clear that in leaves, ascorbate, in collaboration with the thiol system, has a role in protection against reactive oxygen species produced during photosynthesis [212]. Also, thylakoid lumen ascorbate is required for photoprotection *via* VDE activity (Section 7) and it can act as a electron donor to the photosystems when their function is damaged by stress [213], [214]. Outside controlled laboratory environments light intensity is higher, light and temperature fluctuate unpredictably in the short term, mineral nutrients and water supply vary and pests and pathogens are always present. Such conditions have perhaps selected for maintenance of a larger ascorbate pool than is needed for growth in benign conditions. However, an upper limit on ascorbate accumulation might be imposed by removal of H_2_O_2_ or radicals needed as signals to maintain defences and growth. More specifically, ascorbate deficient *vtc* mutants have increased resistance to biotrophic pathogens such as *Pseudomonas syringae.* This is probably caused by increased H_2_O_2_ in the mutants and mediated by salicylic acid, a hormone involved in basal immunity [215], [216], [217], [218], [219]. Conversely, low ascorbate increases sensitivity to *Alternaria*, a necrotrophic pathogen [220]. The upshot is that leaves may need to control ascorbate pool size to balance susceptibility to photo-oxidative stress and necrotrophic *versus* biotrophic pathogens.

## 10 Conclusions: what are the key functions of ascorbate and is it involved in signalling?

Ascorbate has a varied chemistry, enabling the following functions.

1. Detoxification of radicals *via* one electron reduction with formation of the unreactive and “harmless” MDHA radical.
2. Removal of H_2_O_2_ in organisms possessing ascorbate peroxidase (*e.g.* photosynthetic eukaryotes). H_2_O_2_ is produced by oxygen photoreduction at PSI during photosynthetic electron transport and by photorespiration. This provides an additional H_2_O_2_ burden in photosynthetic organisms.
3. Two other plant enzymes require ascorbate. Violaxanthin de-epoxidase (VDE), an essential enzyme in photoprotection of photosynthesis, uses ascorbate as reductant. Myrosinase, a thioglucosidase present in Cruciferous species that synthesise glucosinolates, uses ascorbate in its catalytic mechanism.
4. Reduction of Fe^3+^ to facilitate Fe uptake.
5. Protect 2-oxoglutarate-dependent dioxygenases (2-ODDs) from inactivation by reducing active centre Fe. Impaired collagen synthesis is the most obvious symptom of scurvy. Recently, the recognition that 2-ODDs participate in histone and DNA modifications have resulted in the suggestion that ascorbate could “regulate” epigenetic control of gene expression.

Consistent with the above functions, ascorbate concentration is high in tissues in which dealing with a high flux of oxidants and free radicals, or where defence is critical. Examples are phagocytes (*e.g.* neutrophils), neurons and associated cells, the eye and photosynthetic cells. Consistent with a role in removing radicals, smokers in general require a higher daily intake of ascorbate to maintain its plasma concentration compared to non-smokers. Ascorbate is also high in the adrenal gland, which houses numerous 2-ODD-dependent biosynthetic pathways. These biochemical functions for ascorbate are clear but it is less clear if it can be considered to have a role in signalling, except in a very wide sense. If ascorbate has a direct role in signalling, then a sensing system is required and currently there is no compelling evidence for one. In plants, the closest is the hypothetical ascorbate-binding peptide that controls *VTC2* expression [221]. *E. coli* has an ascorbate 6-P binding protein which controls its ascorbate catabolism operon [61]. The possibility that dehydroascorbylation of proteins by DHA (or its degradation products) could act as a regulatory post-translational modification was reviewed in an earlier section. An example is a mammalian protein kinase involved in NF-κB signalling which is inhibited by DHA binding [222]. These examples are very limited but, given that ascorbate status affects gene expression in both plants and mammals, it is more likely it does so by influencing H_2_O_2_ [159] and free radical levels, which then affect thiol-based signalling [160].

## References

[1] Drouin G., Godin J.-R., Pagé B. (2011). The genetics of vitamin C loss in vertebrates. Curr. Genom..

[2] Wheeler G.L., Ishikawa T., Pornsaksit V., Smirnoff N. (2015). Evolution of alternative biosynthetic pathways for vitamin C following plastid acquisition in photosynthetic eukaryotes. Elife.

[3] Michels A.J., Frei B. (2013). Myths, artifacts, and fatal flaws: identifying limitations and opportunities in vitamin C research. Nutrients.

[4] Halliwell B. (2003). Oxidative stress in cell culture: an under-appreciated problem?. FEBS Lett..

[5] Lachapelle M.Y., Drouin G. (2011). Inactivation dates of the human and guinea pig vitamin C genes. Genetica.

[6] Deutsch J.C. (2000). Dehydroascorbic acid. J. Chromatogr. A.

[7] Buettner G.R., Schafer F.Q. (2006). Ascorbate (Vitamin C), its antioxidant chemistry. Free Radic. Biol. Med..

[8] Du J., Cullen J.J., Buettner G.R. (1826). Ascorbic acid: chemistry, biology and the treatment of cancer. Biochim. Biophys. Acta.

[9] Buettner G.R., Jurkiewicz B.A. (1996). Catalytic metals, ascorbate and free radicals: combinations to avoid. Radiat. Res..

[10] Buettner G.R., Schafer F.Q., Asard H., May J., Smirnoff N. (2003). Ascorbate as an antioxidant. Vitamin C: Its Functions and Biochemistry in Animals and Plants.

[11] Li X., Huang J., May J.M. (2003). Ascorbic acid spares α-tocopherol and decreases lipid peroxidation in neuronal cells. Biochem. Biophys. Res. Commun..

[12] May J.M., Li L., Qu Z., Huang J. (2005). Ascorbate uptake and antioxidant function in peritoneal macrophages. Arch. Biochem. Biophys..

[13] Halliwell B., Foyer C.H. (1976). Ascorbic acid, metal ions and the superoxide radical. Biochem. J..

[14] Jackson T.S., Xu A., Vita J.A., Keaney J.F. (1998). Ascorbate prevents the interaction of superoxide and nitric oxide only at very high physiological concentrations. Circ. Res..

[15] Foyer C., Rowell J., Walker D. (1983). Measurement of the ascorbate content of spinach leaf protoplasts and chloroplasts during illumination. Planta.

[16] Streb P., Feierabend J., Bligny R. (1997). Resistance to photoinhibition of photosystem II and catalase and antioxidative protection in high mountain plants. Plant Cell Environ..

[17] Harrison F.E., May J.M. (2009). Vitamin C function in the brain: vital role of the ascorbate transporter SVCT2. Free Radic. Biol. Med..

[18] Gebicki J.M., Nauser T., Domazou A., Steinmann D., Bounds P.L., Koppenol W.H. (2010). Reduction of protein radicals by GSH and ascorbate: potential biological significance. Amino Acids.

[19] Domazou A.S., Koppenol W.H., Gebicki J.M. (2009). Efficient repair of protein radicals by ascorbate. Free Radic. Biol. Med..

[20] Deutsch J.C. (1998). Spontaneous hydrolysis and dehydration of dehydroascorbic acid in aqueous solution. Anal. Biochem..

[21] Polle A., Junkermann W. (1994). Inhibition of apoplastic and symplastic peroxidase activity from Norway spruce by the photooxidant hydroxymethyl hydroperoxide. Plant Physiol..

[22] Maruta T., Sawa Y., Shigeoka S., Ishikawa T. (2016). Diversity and evolution of ascorbate peroxidase functions in chloroplasts: more than just a classical antioxidant enzyme?. Plant Cell Physiol..

[23] Awad J., Stotz H.U., Fekete A., Krischke M., Engert C., Havaux M., Berger S., Mueller M.J. (2015). 2-Cysteine peroxiredoxins and thylakoid ascorbate peroxidase create a water-watercycle that is essential to protect the photosynthetic apparatus under high light stress conditions. Plant Physiol..

[24] Dietz K.-J. (2016). Thiol-based peroxidases and ascorbate peroxidases: why plants rely on multiple peroxidase systems in the photosynthesizing chloroplast?. Mol. Cells.

[25] Heber U., Miyake C., Mano J., Ohno C., Asada K. (1996). Monodehydroascorbate radical detected by electron paramagnetic resonance spectrometry is a sensitive probe of oxidative stress in intact leaves. Plant Cell Physiol..

[26] Monteiro G., Horta B.B., Pimenta D.C., Augusto O., Netto L.E.S. (2007). Reduction of 1-Cys peroxiredoxins by ascorbate changes the thiol-specific antioxidant paradigm, revealing another function of vitamin C. Proc. Natl. Acad. Sci. USA.

[27] Duarte T.L., Lunec J. (2005). Review: when is an antioxidant not an antioxidant? A review of novel actions and reactions of vitamin C. Free Radic. Res..

[28] Suh J., Zhu B.-Z.Z., Frei B. (2003). Ascorbate does not act as a pro-oxidant towards lipids and proteins in human plasma exposed to redox-active transition metal ions and hydrogen peroxide. Free Radic. Biol. Med..

[29] Parsons H.T., Fry S.C. (2012). Oxidation of dehydroascorbic acid and 2,3-diketogulonate under plant apoplastic conditions. Phytochemistry.

[30] Kobayashi T., Nishizawa N.K. (2012). Iron uptake, translocation, and regulation in higher plants. Annu. Rev. Plant Biol..

[31] Grillet L., Ouerdane L., Flis P., Hoang M.T.T., Isaure M.-P., Lobinski R., Curie C., Mari S. (2014). Ascorbate efflux as a new strategy for iron reduction and transport in plants. J. Biol. Chem..

[32] Smirnoff N., Rebeille F., Douce R. (2011). The metabolism and functions of ascorbic acid in plants.

[33] Urzica E.I., Casero D., Yamasaki H., Hsieh S.I., Adler L.N., Karpowicz S.J., Blaby-Haas C.E., Clarke S.G., Loo J. a., Pellegrini M., Merchant S.S. (2012). Systems and trans-system level analysis identifies conserved iron deficiency responses in the plant lineage. Plant Cell.

[34] Badu-Boateng C., Pardalaki S., Wolf C., Lajnef S., Peyrot F., Naftalin R.J. (2017). Labile iron potentiates ascorbate-dependent reduction and mobilization of ferritin iron. Free Radic. Biol. Med..

[35] Lane D.J.R., Richardson D.R. (2014). The active role of vitamin C in mammalian iron metabolism: much more than just enhanced iron absorption!. Free Radic. Biol. Med..

[36] Kesinger N.G., Stevens J.F. (2009). Covalent interaction of ascorbic acid with natural products. Phytochemistry.

[37] Kesinger N.G., Langsdorf B.L., Yokochi A.F., Miranda C.L., Stevens J.F. (2010). Formation of a vitamin C conjugate of acrolein and its paraoxonase-mediated conversion into 5,6,7,8-tetrahydroxy-4-oxooctanal. Chem. Res. Toxicol..

[38] Sowell J., Conway H.M., Bruno R.S., Traber M.G., Frei B., Stevens J.F. (2005). Ascorbylated 4-hydroxy-2-nonenal as a potential biomarker of oxidative stress response. J. Chromatogr. B Anal. Technol. Biomed. Life Sci..

[39] Gallice P., Sazzarin F., Polverelli M., Cadet J., Berland Y., Crevat A. (1994). Ascorbic acid-2-0-β-glucuronide, a new metabolite of vitamin C identified in human urine and uremic plasma. Biochim. Biophys. Acta.

[40] Kliebenstein D.J., Lambrix V.M., Reichelt M., Gershenzon J., Mitchell-Olds T. (2001). Gene duplication in the diversification of secondary metabolism: tandem 2-oxoglutarate–dependent dioxygenases control glucosinolate biosynthesis in Arabidopsis. Plant Cell.

[41] Opietnik M., Syed Jaafar S., Becker M., Bohmdorfer S., Hofinger A., Rosenau T. (2012). Ascorbigen – Occurrence, synthesis, and analytics. Mini Rev. Org. Chem..

[42] Shikita M., Fahey J.W., Golden T.R., Holtzclaw W.D., Talalay P. (1999). An unusual case of “uncompetitive activation” by ascorbic acid: purification and kinetic properties of a myrosinase from *Raphanus sativus* seedlings. Biochem. J..

[43] Hasenkopf K., Rönner B., Hiller H., Pischetsrieder M. (2002). Analysis of glycated and ascorbylated proteins by gas chromatography-mass spectrometry. J. Agric. Food Chem..

[44] Kay P., Wagner J.R., Gagnon H., Day R., Klarskov K. (2013). Modification of peptide and protein cysteine thiol groups by conjugation with a degradation product of ascorbate. Chem. Res. Toxicol..

[45] Szarka A., Lőrincz T. (2013). The role of ascorbate in protein folding. Protoplasma.

[46] Flandrin A., Allouche S., Rolland Y., McDuff F.O., Richard Wagner J., Klarskov K. (2015). Characterization of dehydroascorbate-mediated modification of glutaredoxin by mass spectrometry. J. Mass Spectrom..

[47] Lin L.S., Varner J.E. (1991). Expression of ascorbic acid oxidase in zucchini squash (*Cucurbita pepo* L.). Plant Physiol..

[48] Raza A., Vince R. (2011). Dehydroascorbic acid adducts of guanosine residues: possible biological implications. ChemBioChem.

[49] Fan X., Monnier V.M. (2008). Inhibition of crystallin ascorbylation by nucleophilic compounds in the hSVCT2 mouse model of lenticular aging. Investig. Ophthalmol. Vis. Sci..

[50] Regulus P., Desilets J.-F., Klarskov K., Wagner J.R. (2010). Characterization and detection in cells of a novel adduct derived from the conjugation of glutathione and dehydroascorbate. Free Radic. Biol. Med..

[51] Bond A.D., Mcclelland B.W., Einstein J., Finamore F.J. (1972). Ascorbic acid-2-sulfate of the brine shrimp *Artemia salina*. Arch. Biochem. Biophys..

[52] Dabrowski K. (1991). Some aspects of ascorbate metabolism in developing embryos of the brine shrimp (*Artemia salina*). Can. J. Fish. Aquat. Sci..

[53] Hancock R.D., Chudek J.A., Walker P.G., Pont S.D.A., Viola R. (2008). Ascorbic acid conjugates isolated from the phloem of Cucurbitaceae. Phytochemistry.

[54] Toyoda-Ono Y., Maeda M., Nakao M., Yoshimura M., Sugiura-Tomimori N., Fukami H. (2004). 2-O-(β-D-Glucopyranosyl)ascorbic acid, a novel ascorbic acid analogue isolated from Lycium fruit. J. Agric. Food Chem..

[55] Spickett C.M., Smirnoff N., Pitt A.R. (2000). The biosynthesis of erythroascorbate in *Saccharomyces cerevisiae* and its role as an antioxidant. Free Radic. Biol. Med..

[56] Kim S., Huh W., Kim J., Hwang S., Kang S. (1996). D-Arabinose dehydrogenase and biosynthesis of erythroascorbic acid in *Candida albicans*. Biochim. Biophys. Acta.

[57] Loewus F.A. (1999). Biosynthesis and metabolism of ascorbic acid in plants and of analogs of ascorbic acid in fungi. Phytochemistry.

[58] Baroja-Mazo A., del Valle P., Rúa J., de Cima S., Busto F., de Arriaga D., Smirnoff N. (2005). Characterisation and biosynthesis of D-erythroascorbic acid in *Phycomyces blakesleeanus*. Fungal Genet. Biol..

[59] Hancock R.D., Galpin J.R., Viola R. (2000). Biosynthesis of L-ascorbic acid (vitamin C) by *Saccharomyces cerevisiae*. FEMS Microbiol. Lett..

[60] Okamura M. (1994). Distribution of ascorbic acid analogs associated glycosides in mushrooms. J. Nutr. Sci. Vitaminol..

[61] Garces F., Fernández F.J., Gómez A.M., Pérez-Luque R., Campos E., Prohens R., Aguilar J., Baldomà L., Coll M., Badía J., Vega M.C. (2008). Quaternary structural transitions in the DeoR-type repressor ular control transcriptional readout from the L-ascorbate utilization regulon in *Escherichia coli*. Biochemistry.

[62] Smirnoff N., Conklin P.L., Loewus F.A. (2001). Biosynthesis of ascorbic acid in plants: a renaissance. Annu. Rev. Plant Physiol. Plant Mol. Biol..

[63] Patananan A.N., Budenholzer L.M., Pedraza M.E., Torres E.R., Adler L.N., Clarke S.G. (2015). The invertebrate *Caenorhabditis elegans* biosynthesizes ascorbate. Arch. Biochem. Biophys..

[64] Linster C.L., Van Schaftingen E. (2007). Vitamin C: biosynthesis, recycling and degradation in mammals. FEBS J..

[65] Bulley S., Laing W. (2016). The regulation of ascorbate biosynthesis. Curr. Opin. Plant Biol..

[66] Smirnoff N., Wheeler G.L. (2000). Ascorbic acid in plants: biosynthesis and function. Crit. Rev. Biochem. Mol. Biol..

[67] Ishikawa T., Nishikawa H., Gao Y., Sawa Y., Shibata H., Yabuta Y., Maruta T., Shigeoka S. (2008). The pathway via D-galacturonate/L-galactonate is significant for ascorbate biosynthesis in *Euglena gracilis*: identification and functional characterization of aldonolactonase. J. Biol. Chem..

[68] Kondo Y., Inai Y., Sato Y., Handa S., Kubo S., Shimokado K., Goto S., Nishikimi M., Maruyama N., Ishigami A. (2006). Senescence marker protein 30 functions as gluconolactonase in L-ascorbic acid biosynthesis, and its knockout mice are prone to scurvy. Proc. Natl. Acad. Sci. USA.

[69] Ames B.N., Cathcart R., Schwiers E., Hochstein P. (1981). Uric acid provides an antioxidant defense in humans against oxidant- and radical-caused aging and cancer: a hypothesis. Proc. Natl. Acad. Sci. USA.

[70] Yoshimura K., Nakane T., Kume S., Shiomi Y., Maruta T., Ishikawa T., Shigeoka S. (2014). Transient expression analysis revealed the importance of *VTC2* expression level in light/dark regulation of ascorbate biosynthesis in *Arabidopsis*. Biosci. Biotechnol. Biochem..

[71] Page M., Sultana N., Paszkiewicz K., Florance H., Smirnoff N. (2012). The influence of ascorbate on anthocyanin accumulation during high light acclimation in Arabidopsis thaliana: further evidence for redox control of anthocyanin synthesis. Plant. Cell Environ..

[72] Dowdle J., Ishikawa T., Gatzek S., Rolinski S., Smirnoff N. (2007). Two genes in *Arabidopsis thaliana* encoding GDP-L-galactose phosphorylase are required for ascorbate biosynthesis and seedling viability. Plant J..

[73] Yabuta Y., Mieda T., Rapolu M., Nakamura A., Motoki T., Maruta T., Yoshimura K., Ishikawa T., Shigeoka S. (2007). Light regulation of ascorbate biosynthesis is dependent on the photosynthetic electron transport chain but independent of sugars. J. Exp. Bot..

[74] Gao Y., Badejo A.A., Shibata H., Sawa Y., Maruta T., Shigeoka S., Page M., Smirnoff N., Ishikawa T. (2011). Expression analysis of the *VTC2* and *VTC5* genes encoding GDP-L-galactose phosphorylase, an enzyme involved in ascorbate biosynthesis, in *Arabidopsis thaliana*. Biosci. Biotechnol. Biochem..

[75] Smirnoff N., Rebeille F., Douce R. (2011). Vitamin C: the metabolism and functions of ascorbic acid in plants.

[76] Conklin P.L., DePaolo D., Wintle B., And C.S., Buckenmeyer G. (2013). Identification of Arabidopsis VTC3 as a putative and unique dual function protein kinase::protein phosphatase involved in the regulation of the ascorbic acid pool in plants. J. Exp. Bot..

[77] Urzica E.I., Adler L.N., Page M.D., Linster C.L., Arbing M.A., Casero D., Pellegrini M., Merchant S.S., Clarke S.G. (2012). Impact of oxidative stress on ascorbate biosynthesis in Chlamydomonas *via* regulation of the *VTC2* gene encoding a GDP-L-galactose phosphorylase. J. Biol. Chem..

[78] Vidal-Meireles A., Neupert J., Zsigmond L., Rosado-Souza L., Kovács L., Nagy V., Galambos A., Fernie A.R., Bock R., Tóth S.Z. (2017). Regulation of ascorbate biosynthesis in green algae has evolved to enable rapid stress-induced response *via* the *VTC2* gene encoding GDP-L-galactose phosphorylase. New Phytol..

[79] Nagy V., Vidal-Meireles A., Tengölics R., Rákhely G., Garab G., Kovács L., Tóth S.Z. (2016). Ascorbate accumulation during sulphur deprivation and its effects on photosystem II activity and H_2_ production of the green alga *Chlamydomonas reinhardtii*. Plant. Cell Environ..

[80] Pallanca J.E., Smirnoff N. (2000). The control of ascorbic acid synthesis and turnover in pea seedlings. J. Exp. Bot..

[81] Leferink N.G.H., van den Berg W.A.M., van Berkel W.J.H. (2008). L-Galactono-γ-lactone dehydrogenase from *Arabidopsis thaliana*, a flavoprotein involved in vitamin C biosynthesis. FEBS J..

[82] Hervás M., Bashir Q., Leferink N.G.H., Ferreira P., Moreno-Beltrán B., Westphal A.H., Díaz-Moreno I., Medina M., de la Rosa M.A., Ubbink M., Navarro J.A., van Berkel W.J.H. (2013). Communication between L-galactono-1,4-lactone dehydrogenase and cytochrome c. FEBS J..

[83] Bartoli C.G., Pastori G.M., Foyer C.H. (2000). Ascorbate biosynthesis in mitochondria is linked to the electron transport chain between complexes III and IV. Plant Physiol..

[84] Schertl P., Sunderhaus S., Klodmann J., Grozeff G.E., Bartoli C.G., Braun H.-P. (2012). L-Galactono-1,4-lactone dehydrogenase (GLDH) forms part of three subcomplexes of mitochondrial complex I in *Arabidopsis thaliana*. J. Biol. Chem..

[85] Schimmeyer J., Bock R., Meyer E.H. (2016). L-Galactono-1,4-lactone dehydrogenase is an assembly factor of the membrane arm of mitochondrial complex I in Arabidopsis. Plant Mol. Biol..

[86] Pineau B., Layoune O., Danon A., Paepe R. De (2008). L-Galactono-1,4-lactone dehydrogenase is required for the accumulation of plant respiratory complex I. J. Biol. Chem..

[87] Smirnoff N., Pallanca J.E. (1996). Ascorbate metabolism in relation to oxidative stress. Biochem. Soc. Trans..

[88] Conklin P., Pallanca J., Last R., Smirnoff N. (1997). L-Ascorbic acid metabolism in the ascorbate-deficient Arabidopsis mutant v*tc1*. Plant Physiol..

[89] Truffault V., Fry S.C., Stevens R.G., Gautier H. (2017). Ascorbate degradation in tomato leads to accumulation of oxalate, threonate and oxalyl threonate. Plant J..

[90] Bonifacio A., Carvalho F.E.L., Martins M.O., Lima Neto M.C., Cunha J.R., Ribeiro C.W., Margis-Pinheiro M., Silveira J.A.G. (2016). Silenced rice in both cytosolic ascorbate peroxidases displays pre-acclimation to cope with oxidative stress induced by 3-aminotriazole-inhibited catalase. J. Plant Physiol..

[91] Queval G., Issakidis-Bourguet E., Hoeberichts F.A., Vandorpe M., Gakière B., Vanacker H., Miginiac-Maslow M., Breusegem F. Van, Noctor G. (2007). Conditional oxidative stress responses in the *Arabidopsis* photorespiratory mutant c*at2* demonstrate that redox state is a key modulator of daylength-dependent gene expression, and define photoperiod as a crucial factor in the regulation of H_2_O_2_-induced cell death. Plant J..

[92] Dewhirst R.A., Clarkson G.J.J., Rothwell S.D., Fry S.C. (2017). Novel insights into ascorbate retention and degradation during the washing and post-harvest storage of spinach and other salad leaves. Food Chem..

[93] German Nutrition Society (2015). New reference values for vitamin C intake. Ann. Nutr. Metab..

[94] Usadel B., Bläsing O.E., Gibon Y., Retzlaff K., Höhne M., Günther M., Stitt M. (2008). Global transcript levels respond to small changes of the carbon status during progressive exhaustion of carbohydrates in Arabidopsis rosettes. Plant Physiol..

[95] Green M.A., Fry S.C. (2005). Vitamin C degradation in plant cells *via* enzymatic hydrolysis of 4-O-oxalyl-L-threonate. Nature.

[96] Kärkönen A., Dewhirst R.A., Mackay C.L., Fry S.C. (2017). Metabolites of 2,3-diketogulonate delay peroxidase action and induce non-enzymic H_2_O_2_ generation: potential roles in the plant cell wall. Arch. Biochem. Biophys..

[97] Keates S.E., Tarlyn N.M., Loewus F.A., Franceschi V.R. (2000). L-Ascorbic acid and L-galactose are sources for oxalic acid and calcium oxalate in Pistia stratiotes. Phytochemistry.

[98] Saito K., Loewus F.A. (1992). Conversion of D-glucosone to oxalic acid and L-(+)-tartaric acid in detached leaves of Pelargonium. Phytochemistry.

[99] Nakata P., McConn M. (2007). Isolated *Medicago truncatula* mutants with increased calcium oxalate crystal accumulation have decreased ascorbic acid levels. Plant Physiol. Biochem..

[100] Yu L., Jiang J.Z., Zhang C., Jiang L.R., Ye N.H., Lu Y.S., Yang G.Z., Liu E., Peng C.L., He Z.H., Peng X.X. (2010). Glyoxylate rather than ascorbate is an efficient precursor for oxalate biosynthesis in rice. J. Exp. Bot..

[101] DeBolt S., Cook D.R., Ford C.M. (2006). L-Tartaric acid synthesis from vitamin C in higher plants. Proc. Natl. Acad. Sci. USA.

[102] Debolt S., Melino V., Ford C.M. (2007). Ascorbate as a biosynthetic precursor in plants. Ann. Bot..

[103] Simpson G.L.W., Ortwerth B.J. (2000). The non-oxidative degradation of ascorbic acid at physiological conditions. Biochim. Biophys. Acta – Mol. Basis Dis..

[104] Nemet I., Monnier V.M. (2011). Vitamin C degradation products and pathways in the human lens. J. Biol. Chem..

[105] Braun L., Puskás F., Csala M., Mészáros G., Mandl J., Bánhegyi G. (1997). Ascorbate as a substrate for glycolysis or gluconeogenesis: evidence for an interorgan ascorbate cycle. Free Radic. Biol. Med..

[106] Loewus F.A., Asard H., May J., Smirnoff N. (2003). Ascorbic acid catabolism: breakdown pathways in animals and plants. Vitamin C: Its Functions and Biochemistry in Animals and Plants.

[107] Pallanca J., Smirnoff N. (1999). Ascorbic acid metabolism in pea seedlings. A comparison of D-glucosone, L-sorbosone, and L-galactono-1,4-lactone as ascorbate precursors. Plant Physiol..

[108] Gara L. De, Asard H., May J., Smirnoff N. (2003). Ascorbate and plant growth: from germination to cell death. Vitamin C: Its Functions and Biochemistry in Animals and Plants.

[109] Arrigoni O., De Gara L., Tommasi F., Liso R. (1992). Changes in the ascorbate system during seed development of *Vicia faba* L. Plant Physiol..

[110] Szecowka M., Heise R., Tohge T., Nunes-Nesi A., Vosloh D., Huege J., Feil R., Lunn J., Nikoloski Z., Stitt M., Fernie A.R., Arrivault S. (2013). Metabolic fluxes in an illuminated *Arabidopsis* rosette. Plant Cell.

[111] Franceschi V.R., Tarlyn N.M. (2002). L-Ascorbic acid is accumulated in source leaf phloem and transported to sink tissues in plants. Plant Physiol..

[112] Hancock R.D., McRae D., Haupt S., Viola R. (2003). Synthesis of L-ascorbic acid in the phloem. BMC Plant Biol..

[113] Horemans N., Szarka A., De Bock M., Raeymaekers T., Potters G., Levine M., Banhegyi G., Guisez Y. (2008). Dehydroascorbate and glucose are taken up into *Arabidopsis thaliana* cell cultures by two distinct mechanisms. FEBS Lett..

[114] Foyer C.H., Lelandais M. (1996). A comparison of the relative rates of transport of ascorbate and glucose across the thylakoid, chloroplast and plasmalemma membranes of pea leaf mesophyll cells. J. Plant Physiol..

[115] Rautenkranz A., Li L., Machler F., Martinoia E., Oertli J.J. (1994). Transport of ascorbic and dehydroascorbic acids across protoplast and vacuole membranes isolated from barley (*Hordeum vulgare* L. cv Gerbel) leaves. Plant Physiol..

[116] Parsons H.T., Fry S.C. (2010). Reactive oxygen species-induced release of intracellular ascorbate in plant cell-suspension cultures and evidence for pulsing of net release rate. New Phytol..

[117] Makavitskaya M., Svistunenko D., Navaselsky I., Hryvusevich P., Mackievic V., Rabadanova C., Tyutereva E., Samokhina V., Straltsova D., Sokolik A., Voitsekhovskaja O., Demidchik V. (2018). Novel roles of ascorbate in plants: induction of cytosolic Ca^2+^ signals and efflux from cells *via* anion channels. J. Exp. Bot..

[118] Miyaji T., Kuromori T., Takeuchi Y., Yamaji N., Yokosho K., Shimazawa A., Sugimoto E., Omote H., Ma J.F., Shinozaki K., Moriyama Y. (2015). AtPHT4;4 is a chloroplast-localized ascorbate transporter in *Arabidopsis*. Nat. Commun..

[119] Fernie A.R., Tóth S.Z. (2015). Identification of the elusive chloroplast ascorbate transporter extends the substrate specificity of the PHT family. Mol. Plant.

[120] Szarka A., Horemans N., Bánhegyi G., Asard H. (2004). Facilitated glucose and dehydroascorbate transport in plant mitochondria. Arch. Biochem. Biophys..

[121] Harrison F.E., Dawes S.M., Meredith M.E., Babaev V.R., Li L., May J.M. (2010). Low vitamin C and increased oxidative stress and cell death in mice that lack the sodium-dependent vitamin C transporter SVCT2. Free Radic. Biol. Med..

[122] Rice M.E., Russo-Menna I. (1997). Differential compartmentalization of brain ascorbate and glutathione between neurons and glia. Neuroscience.

[123] Hansen S.N., Tveden-Nyborg P., Lykkesfeldt J. (2014). Does vitamin C deficiency affect cognitive development and function?. Nutrients.

[124] Covarrubias-Pinto A., Acuña A.I., Beltrán F.A., Torres-Díaz L., Castro M.A. (2015). Old things new view: ascorbic acid protects the brain in neurodegenerative disorders. Int. J. Mol. Sci..

[125] Harrison F.E., May J.M. (2009). Vitamin C function in the brain: vital role of the ascorbate transporter SVCT2. Free Radic. Biol. Med..

[126] Harrison F.E., Bowman G.L., Polidori M.C. (2014). Ascorbic acid and the brain: rationale for the use against cognitive decline. Nutrients.

[127] Reiter R.J., Asard H., May J., Smirnoff N. (2003). Ascorbic acid in the central nervous system: uptake, distribution and functions. Vitamin C: Its Functions and Biochemistry in Animals and Plants.

[128] Michels A.J., Hagen T.M., Frei B. (2013). Human genetic variation influences vitamin C homeostasis by altering vitamin C transport and antioxidant enzyme function. Annu. Rev. Nutr..

[129] Monfort A., Wutz A. (2013). Breathing in epigenetic change with vitamin C. EMBO Rep..

[130] Halliwell B. (2003). Oxidative stress in cell culture: an under-appreciated problem?. Cell.

[131] Schwarzländer M., Wagner S., Ermakova Y.G., Belousov V.V., Radi R., Beckman J.S., Buettner G.R., Demaurex N., Duchen M.R., Forman H.J., Fricker M.D., Gems D., Halestrap A.P., Halliwell B., Jakob U., Johnston I.G., Jones N.S., Logan D.C., Morgan B., Müller F.L., Nicholls D.G., Remington S.J., Schumacker P.T., Winterbourn C.C., Sweetlove L.J., Meyer A.J., Dick T.P., Murphy M.P. (2014). The “mitoflash” probe cpYFP does not respond to superoxide. Nature.

[132] Jung C.H., Wells W.W. (1997). Ascorbic acid is a stimulatory cofactor for mitochondrial glycerol-3-phosphate dehydrogenase. Biochem. Biophys. Res. Commun..

[133] Myllyharju J. (2003). Prolyl 4-hydroxylases, the key enzymes of collagen biosynthesis. Matrix Biol..

[134] Myllyla R., Majamaa K., Giinzler V. (1984). Ascorbate is consumed stoichiometrically in the uncoupled reactions catalyzed by prolyl 4-hydroxylase and lysyl hydroxylase. J. Biol. Chem..

[135] Myllyharju J., Kivirikko K.I. (1997). Characterization of the iron- and 2-oxoglutarate-binding sites of human prolyl 4-hydroxylase. EMBO J..

[136] Blaschke K., Ebata K.T., Karimi M.M., Zepeda-Martínez J.A., Goyal P., Mahapatra S., Tam A., Laird D.J., Hirst M., Rao A., Lorincz M.C., Ramalho-Santos M. (2013). Vitamin C induces Tet-dependent DNA demethylation and a blastocyst-like state in ES cells. Nature.

[137] Flashman E., Davies S.L., Yeoh K.K., Schofield C.J. (2010). Investigating the dependence of the hypoxia-inducible factor hydroxylases (factor inhibiting HIF and prolyl hydroxylase domain 2) on ascorbate and other reducing agents. Biochem. J..

[138] Nytko K.J., Maeda N., Schläfli P., Spielmann P., Wenger R.H., Stiehl D.P., Schläfli P., Spielmann P., Wenger R.H., Stiehl D.P., Schläfli P., Spielmann P., Wenger R.H., Stiehl D.P. (2011). Vitamin C is dispensable for oxygen sensing *in vivo*. Blood.

[139] Young J.I., Züchner S., Wang G. (2015). Regulation of the epigenome by vitamin C. Annu. Rev. Nutr..

[140] Miller P.G., Ebert B.J. (2017). Vitamin C regulates stem cells and cancer. Nature.

[141] Cimmino L., Dolgalev I., Wang Y., Yoshimi A., Martin G.H., Wang J., Ng V., Xia B., Witkowski M.T., Mitchell-Flack M., Grillo I., Bakogianni S., Ndiaye-Lobry D., Martín M.T., Guillamot M., Banh R.S., Xu M., Figueroa M.E., Dickins R.A., Abdel-Wahab O., Park C.Y., Tsirigos A., Neel B.G., Aifantis I. (2017). Restoration of TET2 function blocks aberrant self-renewal and leukemia progression. Cell.

[142] Agathocleous M., Meacham C.E., Burgess R.J., Piskounova E., Zhao Z., Crane G.M., Cowin B.L., Bruner E., Murphy M.M., Chen W., Spangrude G.J., Hu Z., DeBerardinis R.J., Morrison S.J. (2017). Ascorbate regulates haematopoietic stem cell function and leukaemogenesis. Nature.

[143] Mellor N., Band L.R., Pěnčík A., Novák O., Rashed A., Holman T., Wilson M.H., Voß U., Bishopp A., King J.R., Ljung K., Bennett M.J., Owen M.R. (2016). Dynamic regulation of auxin oxidase and conjugating enzymes *AtDAO1* and *GH3* modulates auxin homeostasis. Proc. Natl. Acad. Sci. USA.

[144] Kliebenstein D.J., Lambrix V., Reichelt M., Gershenzon J., Mitchell-Olds T. (2001). Gene duplication in the diversification of secondary metabolism: tandem 2-oxoglutarate-dependent dioxygenases control glucosinolate biosynthesis in Arabidopsis. Plant Cell.

[145] Brisson L., El Bakkali-Taheri N., Giorgi M., Fadel A., Kaizer J., Réglier M., Tron T., Ajandouz E.H., Simaan A.J. (2012). 1-Aminocyclopropane-1-carboxylic acid oxidase: insight into cofactor binding from experimental and theoretical studies. J. Biol. Inorg. Chem..

[146] White M.D., Klecker M., Hopkinson R.J., Weits D.A., Mueller C., Naumann C., O’Neill R., Wickens J., Yang J., Brooks-Bartlett J.C., Garman E.F., Grossmann T.N., Dissmeyer N., Flashman E. (2017). Plant cysteine oxidases are dioxygenases that directly enable arginyl transferase-catalysed arginylation of N-end rule targets. Nat. Commun..

[147] Nguyen-Kim H., San Clemente H., Balliau T., Zivy M., Dunand C., Albenne C., Jamet E. (2016). Arabidopsis thaliana root cell wall proteomics: increasing the proteome coverage using a combinatorial peptide ligand library and description of unexpected Hyp in peroxidase amino acid sequences. Proteomics.

[148] Sultana N., Florance H.V., Johns A., Smirnoff N. (2015). Ascorbate deficiency influences the leaf cell wall glycoproteome in *Arabidopsis thaliana*. Plant Cell Environ..

[149] Vanderauwera S., Zimmermann P., Rombauts S., Vandenabeele S., Langebartels C., Gruissem W., Inzé D., Breusegem F. Van (2005). Genome-wide analysis of hydrogen peroxide-regulated gene expression in Arabidopsis reveals a high light-induced transcriptional cluster involved in anthocyanin biosynthesis. Plant Physiol..

[150] Saga G., Giorgetti A., Fufezan C., Giacometti G.M., Bassi R., Morosinotto T. (2010). Mutation analysis of violaxanthin de-epoxidase identifies substrate-binding sites and residues involved in catalysis. J. Biol. Chem..

[151] Kromdijk J., Głowacka K., Leonelli L., Gabilly S.T., Iwai M., Niyogi K.K., Long S.P. (2016). Improving photosynthesis and crop productivity by accelerating recovery from photoprotection. Science.

[152] Müller-Moulé P., Havaux M., Niyogi K.K. (2003). Zeaxanthin deficiency enhances the high light sensitivity of an ascorbate-deficient mutant of Arabidopsis. Plant Physiol..

[153] Mu P., Conklin P.P.L., Niyogi K.K., Müller-Moulé P. (2002). Ascorbate deficiency can limit violaxanthin de-epoxidase activity in vivo. Plant Physiol..

[154] Müller-Moulé P., Golan T., Niyogi K.K. (2004). Ascorbate-deficient mutants of *Arabidopsis* grow in high light despite chronic photooxidative stress. Plant Physiol..

[155] Smirnoff N. (2000). Ascorbate biosynthesis and function in photoprotection. Philos. Trans. R. Soc. Lond. B Biol. Sci..

[156] Maruta T., Sawa Y., Shigeoka S., Ishikawa T. (2016). Diversity and evolution of ascorbate peroxidase functions in chloroplasts: more than just a classical antioxidant enzyme?. Plant Cell Physiol..

[157] Kitajima S., Kitamura M., Koja N. (2008). Triple mutation of Cys26, Trp35, and Cys126 in stromal ascorbate peroxidase confers H_2_O_2_ tolerance comparable to that of the cytosolic isoform. Biochem. Biophys. Res. Commun..

[158] Kitajima S., Nii H., Kitamura M. (2010). Recombinant stromal APX defective in the unique loop region showed improved tolerance to hydrogen peroxide. Biosci. Biotechnol. Biochem..

[159] Exposito-Rodriguez M., Laissue P.P., Yvon-Durocher G., Smirnoff N., Mullineaux P.M. (2017). Photosynthesis-dependent H_2_O_2_ transfer from chloroplasts to nuclei provides a high-light signalling mechanism. Nat. Commun..

[160] Mullineaux P.M., Exposito-Rodriguez M., Laissue P.P., Smirnoff N. (2018). ROS-dependent signaling pathways in plants and algae exposed to high light: comparisons with other eukaryotes. Free Radic. Biol. Med..

[161] Correa-Aragunde N., Foresi N., Delledonne M., Lamattina L. (2013). Auxin induces redox regulation of ascorbate peroxidase 1 activity by S-nitrosylation/denitrosylation balance resulting in changes of root growth pattern in Arabidopsis. J. Exp. Bot..

[162] Correa-Aragunde N., Foresi N., Lamattina L. (2015). Nitric oxide is a ubiquitous signal for maintaining redox balance in plant cells: regulation of ascorbate peroxidase as a case study. J. Exp. Bot..

[163] Yang H., Mu J., Chen L., Feng J., Hu J., Li L., Zhou J.-M., Zuo J. (2015). S -Nitrosylation positively regulates ascorbate peroxidase activity during plant stress responses. Plant Physiol..

[164] Mittler R., Vanderauwera S., Gollery M., Van Breusegem F. (2004). Reactive oxygen gene network of plants. Trends Plant Sci..

[165] Karpinski S., Escobar C., Karpinska B., Creissen G., Mullineaux P.M. (1997). Photosynthetic electron transport regulates the expression of cytosolic ascorbate peroxidase genes in *Arabidopsis* during excess light stress. Plant Cell.

[166] Koussevitzky S., Suzuki N., Huntington S., Armijo L., Sha W., Cortes D., Shulaev V., Mittler R. (2008). Ascorbate peroxidase 1 plays a key role in the response of *Arabidopsis thaliana* to stress combination. J. Biol. Chem..

[167] van Buer J., Cvetkovic J., Baier M. (2016). Cold regulation of plastid ascorbate peroxidases serves as a priming hub controlling ROS signaling in *Arabidopsis thaliana*. BMC Plant Biol..

[168] May J., Asard H., Asard H., May J., Smirnoff N. (2003). Ascorbate recycling. Vitamin C: Its Functions and Biochemistry in Animals and Plants.

[169] Dixon D.P., Davis B.G., Edwards R. (2002). Functional divergence in the glutathione transferase superfamily in plants. Identification of two classes with putative functions in redox homeostasis in *Arabidopsis thaliana*. J. Biol. Chem..

[170] Saitoh Y., Fukuoka Y., Nishikimi M., Miwa N. (2007). Transfection with glutathione-dependent dehydroascorbate reductase genes exerts cytoprotective effects against hydroperoxide-induced cell injury through vitamin C regeneration and oxidative-stress diminishment. Gene Ther. Mol. Biol..

[171] Washburn M.P., Wells W.W. (1999). The catalytic mechanism of the glutathione-dependent dehydroascorbate reductase activity of thioltransferase (glutaredoxin). Biochemistry.

[172] Zhang Y.-J., Wang W., Yang H.-L., Li Y., Kang X.-Y., Wang X.-R., Yang Z.-L. (2015). Molecular properties and functional divergence of the dehydroascorbate reductase gene family in lower and higher plants. PLoS One.

[173] Smirnoff N., Rebeille F., Douce R. (2011). Vitamin C: the metabolism and functions of ascorbic acid in plants.

[174] Do H., Kim I.-S., Jeon B.W., Lee C.W., Park A.K., Wi A.R., Shin S.C., Park H., Kim Y.-S., Yoon H.-S., Kim H.-W., Lee J.H. (2016). Structural understanding of the recycling of oxidized ascorbate by dehydroascorbate reductase (OsDHAR) from *Oryza sativa* L. japonica. Sci. Rep..

[175] Bodra N., Young D., Astolfi Rosado L., Pallo A., Wahni K., De Proft F., Huang J., Van Breusegem F., Messens J. (2017). *Arabidopsis thaliana* dehydroascorbate reductase 2: conformational flexibility during catalysis. Sci. Rep..

[176] Chang H.-Y., Lin S.-T., Ko T.-P., Wu S.-M., Lin T.-H., Chang Y.-C., Huang K.-F., Lee T.-M. (2017). Enzymatic characterization and crystal structure analysis of *Chlamydomonas reinhardtii* dehydroascorbate reductase and their implications for oxidative stress. Plant Physiol. Biochem..

[177] Noshi M., Hatanaka R., Tanabe N., Terai Y., Maruta T., Shigeoka S. (2016). Redox regulation of ascorbate and glutathione by a chloroplastic dehydroascorbate reductase is required for high-light stress tolerance in Arabidopsis. Biosci. Biotechnol. Biochem..

[178] Noshi M., Yamada H., Hatanaka R., Tanabe N., Tamoi M., Shigeoka S. (2017). Arabidopsis dehydroascorbate reductase 1 and 2 modulate redox states of ascorbate-glutathione cycle in the cytosol in response to photooxidative stress. Biosci. Biotechnol. Biochem..

[179] Chen Z., Gallie D.R. (2005). Increasing tolerance to ozone by elevating foliar ascorbic acid confers greater protection against ozone than increasing avoidance. Plant Physiol..

[180] Chen Z., Young T.E., Ling J., Chang S.-C., Gallie D.R. (2003). Increasing vitamin C content of plants through enhanced ascorbate recycling. Proc. Natl. Acad. Sci. USA.

[181] Lin S.T., Chiou C.W., Chu Y.L., Hsiao Y., Tseng Y.F., Chen Y.C., Chen H.J., Chang H.Y., Lee T.M. (2016). Enhanced ascorbate regeneration via dehydroascorbate reductase confers tolerance to photo-oxidative stress in *Chlamydomonas reinhardtii*. Plant Cell Physiol..

[182] Yoshida S., Tamaoki M., Shikano T., Nakajima N., Ogawa D., Ioki M., Aono M., Kubo A., Kamada H., Inoue Y., Saji H. (2006). Cytosolic dehydroascorbate reductase is important for ozone tolerance in *Arabidopsis thaliana*. Plant Cell Physiol..

[183] Ushimaru T., Nakagawa T., Fujioka Y., Daicho K., Naito M., Yamauchi Y., Nonaka H., Amako K., Yamawaki K., Murata N. (2006). Transgenic Arabidopsis plants expressing the rice dehydroascorbate reductase gene are resistant to salt stress. J. Plant Physiol..

[184] Eltelib H.A., Fujikawa Y., Esaka M. (2012). Overexpression of the acerola (*Malpighia glabra*) monodehydroascorbate reductase gene in transgenic tobacco plants results in increased ascorbate levels and enhanced tolerance to salt stress. S. Afr. J. Bot..

[185] Kim Y.S., Kim I.S., Bae M.J., Choe Y.H., Kim Y.H., Park H.M., Kang H.G., Yoon H.S. (2013). Homologous expression of cytosolic dehydroascorbate reductase increases grain yield and biomass under paddy field conditions in transgenic rice (*Oryza sativa* L. japonica). Planta.

[186] Chen Z., Gallie D.R. (2004). The ascorbic acid redox state controls guard cell signaling and stomatal movement. Plant Cell.

[187] Grace S., Pace R., Wydrzynski T. (1995). Formation and decay of monodehydroascorbate radicals in illuminated thylakoids as determined by EPR spectroscopy. Biochim. Biophys. Acta.

[188] Hossain A., Nakano Y., Asada K. (1984). Monodehydroascorbate reductase in spinach chloroplasts and its participation in regeneration of ascorbate for scavenging hydrogen peroxide. Plant Cell Physiol..

[189] Park A.K., Kim I.-S., Do H., Jeon B.W., Lee C.W., Roh S.J., Shin S.C., Park H., Kim Y.-S., Kim Y.-H., Yoon H.-S., Lee J.H., Kim H.-W. (2016). Structure and catalytic mechanism of monodehydroascorbate reductase, MDHAR, from *Oryza sativa* L. japonica. Sci. Rep..

[190] Sakihama Y., Cohen M.F., Grace S.C., Yamasaki H. (2002). Plant phenolic antioxidant and prooxidant activities: phenolics-induced oxidative damage mediated by metals in plants. Toxicology.

[191] Eastmond P.J. (2007). MONODEHYROASCORBATE REDUCTASE4 is required for seed storage oil hydrolysis and postgerminative growth in Arabidopsis. Plant Cell.

[192] Vanacker H., Carver T.L.W., Foyer C.H. (1998). Pathogen-induced changes in the antioxidant status of the apoplast in barley leaves. Plant Physiol..

[193] Sakihama Y., Mano J., Sano S., Asada K., Yamasaki H. (2000). Reduction of phenoxyl radicals mediated by monodehydroascorbate reductase. Biochem. Biophys. Res. Commun..

[194] Johnston E.J., Rylott E.L., Beynon E., Lorenz A., Chechik V., Bruce N.C. (2015). Monodehydroascorbate reductase mediates TNT toxicity in plants. Science.

[195] Kärkönen A., Dewhirst R.A., Mackay C.L., Fry S.C. (2017). Metabolites of 2,3-diketogulonate delay peroxidase action and induce non-enzymic H_2_O_2_ generation: potential roles in the plant cell wall. Arch. Biochem. Biophys..

[196] Sanmartin M., Drogoudi P.D., Lyons T., Pateraki I., Barnes J., Kanellis A.K. (2003). Over-expression of ascorbate oxidase in the apoplast of transgenic tobacco results in altered ascorbate and glutathione redox states and increased sensitivity to ozone. Planta.

[197] Pignocchi C., Fletcher J.M., Wilkinson J.E., Barnes J.D., Foyer C.H. (2003). The function of ascorbate oxidase in tobacco. Plant Physiol..

[198] Garchery C., Gest N., Do P.T., Alhagdow M., Baldet P., Menard G., Rothan C., Massot C., Gautier H., Aarrouf J., Fernie A.R., Stevens R. (2013). A diminution in ascorbate oxidase activity affects carbon allocation and improves yield in tomato under water deficit. Plant. Cell Environ..

[199] Yamamoto A., Bhuiyan M.N.H., Waditee R., Tanaka Y., Esaka M., Oba K., Jagendorf A.T., Takabe T. (2005). Suppressed expression of the apoplastic ascorbate oxidase gene increases salt tolerance in tobacco and Arabidopsis plants. J. Exp. Bot..

[200] Pignocchi C. (2003). Apoplastic ascorbate metabolism and its role in the regulation of cell signalling. Curr. Opin. Plant Biol..

[201] De Tullio M., Guether M., Balestrini R. (2013). Ascorbate oxidase is the potential conductor of a symphony of signaling pathways. Plant Signal. Behav..

[202] Foster S.J., Asensi A., Taybi T., Pignocchi C., Kiddle G., Herna I., Barnes J., Foyer C.H. (2006). Ascorbate oxidase-dependent changes in the redox state of the apoplast modulate gene transcript accumulation leading to modified hormone signaling and orchestration of defense processes in tobacco. Plant Physiol..

[203] Esaka M., Fujisawa K., Goto M., Kisu Y. (1992). Regulation of ascorbate oxidase expression in pumpkin by auxin and copper. Plant Physiol..

[204] Fotopoulos V., De Tullio M.C., Barnes J., Kanellis A.K. (2008). Altered stomatal dynamics in ascorbate oxidase over-expressing tobacco plants suggest a role for dehydroascorbate signalling. J. Exp. Bot..

[205] Dowdle J., Ishikawa T., Gatzek S., Rolinski S., Smirnoff N. (2007). Two genes in Arabidopsis thaliana encoding GDP-L-galactose phosphorylase are required for ascorbate biosynthesis and seedling viability. Plant J..

[206] Lim B., Smirnoff N., Cobbett C.S., Golz J.F. (2016). Ascorbate-deficient v*tc2* mutants in Arabidopsis do not exhibit decreased growth. Front. Plant Sci..

[207] Kerchev P.I., Pellny T.K., Vivancos P.D., Kiddle G., Hedden P., Driscoll S., Vanacker H., Verrier P., Hancock R.D., Foyer C.H. (2011). The transcription factor ABI4 Is required for the ascorbic acid-dependent regulation of growth and regulation of jasmonate-dependent defense signaling pathways in *Arabidopsis*. Plant Cell.

[208] Conklin P.L., Williams E.H., Last R.L. (1996). Environmental stress sensitivity of an ascorbic acid-deficient Arabidopsis mutant. Proc. Natl. Acad. Sci. USA.

[209] Filkowski J., Kovalchuk O., Kovalchuk I. (2004). Genome stability of v*tc1, tt4,* and *tt5 Arabidopsis thaliana* mutants impaired in protection against oxidative stress. Plant J..

[210] Genty B., Havaux M., Triantaphylide C. (2006). Autoluminescence imaging: a non-invasive tool for mapping oxidative stress. Trends Plant Sci..

[211] Müller-Moulé P., Havaux M., Niyogi K.K. (2003). Zeaxanthin deficiency enhances the high light sensitivity of an ascorbate-deficient mutant of Arabidopsis. Plant Physiol..

[212] Awad J., Smin, Stotz H.U., Fekete A., Krischke M., Engert C., Havaux M., Berger S., Mueller M.J. (2015). 2-Cysteine peroxiredoxins and thylakoid ascorbate peroxidase create a water-watercycle that is essential to protect the photosynthetic apparatus under high light stress conditions. Plant Physiol..

[213] Tóth S.Z., Puthur J.T., Nagy V., Garab G. (2009). Experimental evidence for ascorbate-dependent electron transport in leaves with inactive oxygen-evolving complexes. Plant Physiol..

[214] Tóth S.Z., Nagy V., Puthur J.T., Kovács L., Garab G. (2011). The physiological role of ascorbate as photosystem II electron donor: protection against photoinactivation in heat-stressed leaves. Plant Physiol..

[215] Veljovic-Jovanovic S.D., Pignocchi C., Noctor G., Foyer C.H. (2001). Low ascorbic acid in the v*tc1* mutant of Arabidopsis is associated with decreased growth and intracellular redistribution of the antioxidant system. Plant Physiol..

[216] Pavet V., Olmos E., Kiddle G., Mowla S., Kumar S., Antoniw J., Alvarez M.E., Foyer C.H. (2005). Ascorbic acid deficiency activates cell death and disease resistance responses in Arabidopsis. Plant Physiol..

[217] Colville L., Smirnoff N. (2008). Antioxidant status, peroxidase activity, and PR protein transcript levels in ascorbate-deficient *Arabidopsis thaliana vtc* mutants. J. Exp. Bot..

[218] Mukherjee M., Larrimore K.E., Ahmed N.J., Bedick T.S., Barghouthi N.T., Traw M.B., Barth C. (2010). Ascorbic acid deficiency in *Arabidopsis* induces constitutive priming that is dependent on hydrogen peroxide, salicylic acid, and the *NPR1* gene. Mol. Plant-Microbe Interact..

[219] Barth C., Moeder W., Klessig D.F., Conklin P.L. (2004). The timing of senescence and response to pathogens is altered in the ascorbate-deficient Arabidopsis mutant *vitamin c-1*. Plant Physiol..

[220] Botanga C.J., Bethke G., Chen Z., Gallie D.R., Fiehn O., Glazebrook J. (2012). Metabolite profiling of Arabidopsis inoculated with *Alternaria brassicicola* reveals that ascorbate reduces disease severity. Mol. Plant-Microbe Interact..

[221] Laing W.A., Martínez-Sánchez M., Wright M.A., Bulley S.M., Brewster D., Dare A.P., Rassam M., Wang D., Storey R., Macknight R.C., Hellens R.P. (2015). An upstream open reading frame is essential for feedback regulation of ascorbate biosynthesis in Arabidopsis. Plant Cell.

[222] Carcamo J.M., Pedraza A., Borquez-Ojeda O., Zhang B., Sanchez R., Golde D.W. (2004). Vitamin C is a kinase inhibitor: dehydroascorbic acid inhibits IκBα Kinase β. Mol. Cell. Biol..

